# A Review on Nano-/Microstructured Materials Constructed by Electrochemical Technologies for Supercapacitors

**DOI:** 10.1007/s40820-020-00451-z

**Published:** 2020-05-30

**Authors:** Huizhen Lv, Qing Pan, Yu Song, Xiao-Xia Liu, Tianyu Liu

**Affiliations:** 1 Department of Chemistry, Northeastern University, Shenyang, 110819 People’s Republic of China; 2 Department of Chemistry, Virginia Polytechnic Institute and State University, Blacksburg, VA 24061 USA

**Keywords:** Nanostructure, Microstructure, Electrochemical, Synthesis, Supercapacitor

## Abstract

The article reviews the recent progress of electrochemical techniques on synthesizing nano-/microstructures as supercapacitor electrodes. With a history of more than a century, electrochemical techniques have evolved from metal plating since their inception to versatile synthesis tools for electrochemically active materials of diverse morphologies, compositions, and functions. The review begins with tutorials on the operating mechanisms of five commonly used electrochemical techniques, including cyclic voltammetry, potentiostatic deposition, galvanostatic deposition, pulse deposition, and electrophoretic deposition, followed by thorough surveys of the nano-/microstructured materials synthesized electrochemically. Specifically, representative synthesis mechanisms and the state-of-the-art electrochemical performances of exfoliated graphene, conducting polymers, metal oxides, metal sulfides, and their composites are surveyed. The article concludes with summaries of the unique merits, potential challenges, and associated opportunities of electrochemical synthesis techniques for electrode materials in supercapacitors.

## Highlights

- Recent progress of active materials in supercapacitors synthesized by electrochemical techniques is reviewed.
- Electrochemically synthesized nanostructures of various dimensions, compositions, and electrochemical properties are discussed.
- The advantages and challenges of electrochemical technologies in preparing nano-/microstructured materials for electrochemical energy storage devices are summarized.

## Graphical abstract

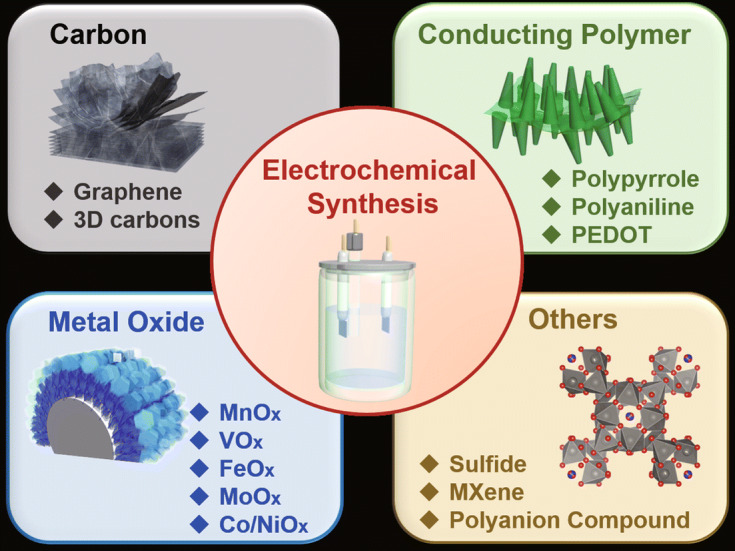

## Introduction

The rapidly expanding markets of mobile electronics, electrified transportation, wireless networks (e.g., the Internet of Things), and sustainable energy utilization have substantially fueled the development of electrochemical energy storage systems [1–4]. Supercapacitors, including electrochemical capacitors and pseudocapacitors, stand out among diverse arrays of energy storage devices due to their ultrahigh power density and ultralong life spans [5–9]. Since the first introduction in 1957 by Howard Becker of American General Electric [10], supercapacitors have pervaded in applications demanding energy input and output with high power, e.g., the power source to the emergency doors of Airbus A380 aircraft [11, 12]. Therefore, elevating the capacitance and energy density of supercapacitors at ultrafast charging rates has remained a central topic [13–16]. Extensive research efforts have been devoted to developing high-performance electrode materials [13, 15–17], since electrodes primarily determine the capacitance, energy density, and power density of supercapacitors.

Supercapacitors are classified into two categories: electric double-layer capacitors (EDLCs) and pseudocapacitors. EDLCs store charges through adsorption and desorption of ions in electrolytes at the electrolyte/electrode interfaces. Carbon materials are conventional electrode materials in EDLCs. Pseudocapacitors store charges through kinetically fast Faradaic reactions. Surface redox pseudocapacitance, intercalation pseudocapacitance, and underpotential deposition are examples of pseudocapacitance [13]. Redox pseudocapacitance occurs when electrolyte ions adsorbed on or near the electrode surfaces and involves interfacial charge transfer. Intercalation pseudocapacitance comes from reversible insertion and desertion of electrolyte ions in layered or tunneled electrode materials without phase transitions. Underpotential deposition will be elaborated in Sect. 2.2, but it is not used for charge storage due to its limited capacity. Pseudocapacitance has its electrochemical features, such as quasi-rectangular cyclic voltammetry curves, linear galvanostatic charge/discharge profiles, as well as a near-linear response between current and scan rate in cyclic voltammograms. These characteristics must be clearly distinguished from battery-type behaviors (Sect. 5.6).

Electrochemical techniques are a group of synthesis methods of versatile active materials in supercapacitors. These methods have a history of more than 150 years [18], and their first application is to plate metals for jewelry decoration, surface protection, and electronic-circuit manufacturing [19]. Along with the boom of electrochemical energy storage, the role of electrochemical techniques has been enriched by synthesizing nano-/microstructured materials for electrochemical energy storage [2, 20]. The syntheses involve electrochemical processes including reduction, oxidation, gas evolution, ion intercalation, and combinations of these methods thereof. Chemical reactions, such as the electrolysis of electrolytes (e.g., H_2_ and O_2_ evolution in aqueous electrolytes), redox reactions of electrolyte ions, as well as modifications of the structures and compositions of electrode materials, are typically accompanying phenomena.

Compared to other synthesis strategies, electrochemical techniques have their unique advantages. First, they are facile and mild. Room temperature, ambient pressure, and aqueous solutions are sufficient for performing electrochemical techniques. Second, the experimental setups, such as electrolytic cells and electrochemical workstations, are often readily available in electrochemistry laboratories. This availability allows electrochemists to prepare active materials without delicate instruments and sophisticated protocols. Perhaps the most striking feature of electrochemical techniques is their high tunability toward the structure, composition, property, and morphology of products [2, 20]. Magnitudes of applied current or voltage, types and concentrations of salts in electrolytes, reaction durations, solution temperatures, as well as substrate morphologies are all tunable parameters that result in versatile materials. Benefited from these merits, electrochemical techniques have been extensively studied and rapidly developed in the past decades. The materials that have been prepared electrochemically include exfoliated graphene [21], metal oxides [22–24], conducting polymers [25, 26], and their composites [27–29].

This review article presents a thorough survey of electrochemically synthesized nano-/microstructured materials for supercapacitors. It starts with introduction of the operating mechanisms, characteristics of input and output signals, strengths, and weaknesses of cyclic voltammetry, potentiostatic (constant voltage) and galvanostatic (constant current) depositions, pulse electrodeposition, and electrophoretic deposition. Afterward, the article reviews the recent progress of the active materials synthesized by electrochemical techniques. This part is segmented based on the compositions of materials, including carbon-based materials, metal oxides, conducting polymers, composites, and other materials. Each subsection starts with typical synthesis mechanisms and is exemplified with one or more representative examples in the literature. At last, the article comments on the merits, challenges, and opportunities of electrochemical technologies in terms of synthesizing nano-/microstructured materials for electrochemical energy storage.

## Fundamentals of Electrochemical Synthesis Techniques

**Fig. 1:**
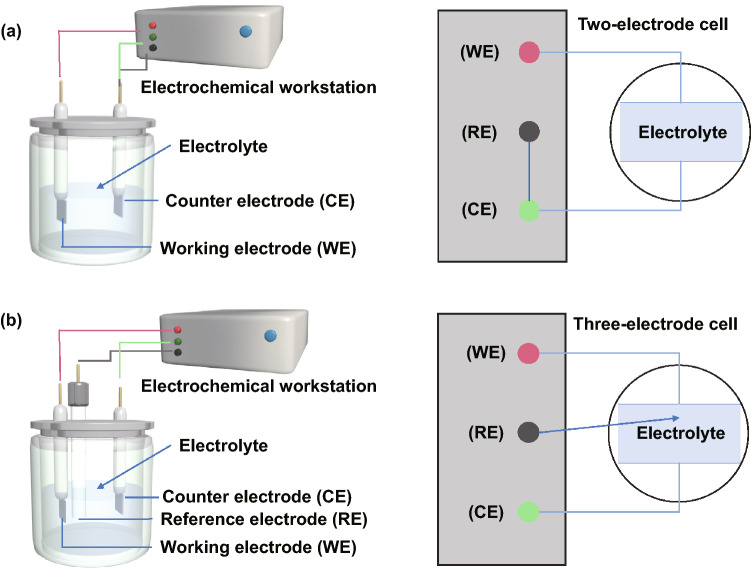
Schemes illustrating the experimental setups of **a** two-electrode and **b** three-electrode electrolytic cells for electrochemical syntheses

Cyclic voltammetry, galvanostatic deposition, potentiostatic deposition, pulse deposition, and electrophoretic deposition constitute the most widely and extensively investigated and practiced electrochemical synthesis techniques for nano-/microstructured materials as supercapacitor electrodes. These processes are typically carried out in electrolytic cells powered by electrochemical workstations. Based on the number of electrodes involved, the setup for electrochemical synthesis is categorized into two types: two-electrode (Fig. 1a) and three-electrode (Fig. 1b) configurations [30]. The two-electrode configuration contains a positive electrode and a negative electrode that are both immersed in electrolytes. An electrochemical workstation or power source provides voltage across the two electrodes. Therefore, the measured voltage in this scenario is the overall cell voltage. The three-electrode system comprises a working electrode (WE), a counter electrode (CE), and a reference electrode (RE). Ideally, current flows only between WE and CE, and the voltage of WE is referenced to that of RE. Saturated calomel electrode (SCE), Ag/AgCl, and Hg/HgO electrodes with their nearly constant half-reaction potentials are common REs. REs are placed in vicinity to WEs to minimize *iR* drop and voltage fluctuation due to electrolyte resistance [31]. The measured voltage in three-electrode configurations is the real-time potential of WEs.

### Cyclic Voltammetry (CV)

Besides a conventional electrochemical technique for probing electrochemically redox activities [32], CV serves as a synthetic tool. It linearly scans potential within a range, termed potential window, and simultaneously records current as a response [33]. Increasing the applied potential, the forward scan oxidizes species in electrolytes or on electrodes and produces anodic current. Conversely, the backward scan decreases the applied potential, reduces active components, and generates a cathodic current.

**Fig. 2:**
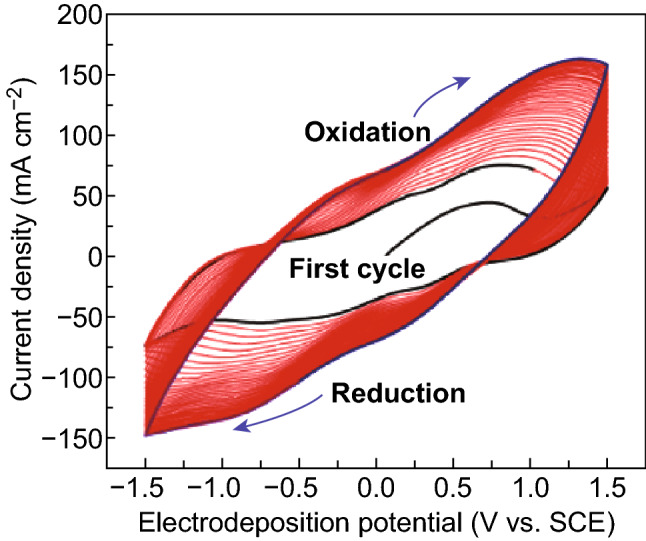
CV curves of the deposition of mixed-valence vanadium oxide nanorods

CV has three main advantages as a synthetic approach. First, it allows for determining the onset potential of an electrodeposition reaction. Oxidation or reduction reactions involving charge transfer across the electrolyte–electrode interfaces will display sharp increases or well-defined peaks in the current. Since the onset potential is the minimum voltage needed to initiate electrodeposition reactions, CV is useful for developing experimental protocols. Second, the potential linear scan of CV is beneficial for growing uniform and conformal films. This characteristic provides a gradient driving force for deposition: Deposition will only begin until the potential is scanned above the onset potential, and the driving force of deposition scales linearly with the potential gradually elevated away from the onset potential. This gradient driving force of CV adjusts the deposition rate and avoids consistently high deposition voltages that can lead to overgrowth of materials, rapid clogging of pores, and/or uneven deposition of films. Third, CV is suitable for synthesizing materials with multiple valence states, e.g., transition metal oxides. For example, deposited by CV within a potential range between − 1.5 and 1.5 V vs. SCE in a 0.1 M VOSO_4_ aqueous electrolyte, vanadium oxide nanorods contained ~ 50% V_5_O_12_ (a mixture of V^5+^ and V^4+^) and ~ 50% VO_2_ [34]. The high valence state, V^5+^, formed during the anodic or forward scan, while the as-deposited V_5_O_12_ was partially reduced to VO_2_ in the subsequent cathodic or backward scan. These redox processes were documented by the broad peaks in the corresponding CV curve (Fig. 2). Compounds with multivalent species reportedly possess augmented capacitance [35], improved rate capability [34, 36], as well as enhanced cycling stability [37, 38] compared to their monovalent counterparts.

### Potentiostatic (Constant Voltage) Deposition

**Fig. 3:**
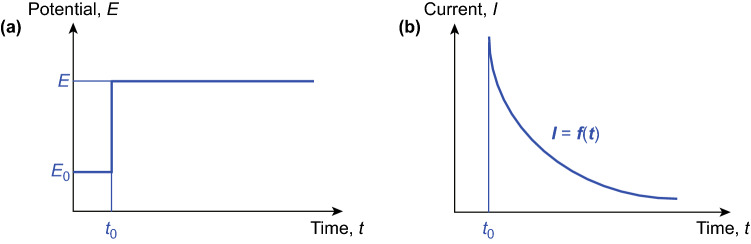
**a** Constant potential applied in potentiostatic deposition; *E*_0_ and *E* are the open-circuit potential and the applied potential; *t*_0_ marks the starting time of deposition. **b** Corresponding current as a function of time

Potentiostatic deposition synthesizes materials by applying a constant potential across the positive and negative electrodes (two-electrode system) or between the working and counter electrodes (three-electrode system). The deposition potential is maintained constant by an electrochemical workstation (Fig. 3a), and the current is recorded as a function of time (Fig. 3b) [39]. Based on the difference between applied and thermodynamic equilibrium potentials, potentiostatic deposition is categorized into underpotential deposition (UPD) and overpotential deposition (OPD) [40, 41].

UPD happens at potentials below thermodynamic equilibrium potentials. For example, metal deposition can initiate at potentials smaller than the corresponding equilibrium reduction potentials, due to the lower work function of the deposited metal than that of the substrate metal, as described by the Kolb–Gerischer equation [42]:

1 $$\Delta E = 0.5\Delta \phi$$

where $\Delta E$ is the downshift of deposition potential (in V) and $\Delta \phi$ is the difference in the work functions between the deposited and substrate metals (in eV). The coefficient 0.5 (in V eV^−1^) comes from a linear fitting involving 21 metal–metal couples [42]. UPD involves adsorption, nucleation, and growth processes determined by surface characteristics of substrates (e.g., chemical composition, crystal structure, morphology, and electrolyte wettability) and ion–substrate interactions. Besides, the types of cations in electrolytes and anions strongly influence the structure and properties of the deposited materials, as well as deposition kinetics [43–45]. One example is the UPD of Cu on Au(111) facets in aqueous sulfuric acid solutions [46]. The deposition was much slower under pH = 2 than pH = 4. This discrepancy in the deposition rate was correlated with the different anions under different pH values. Increasing the solution acidity converted bisulfate ions to sulfate ions. The latter adsorbed much more strongly than the former on the gold substrate, which blocked some active sites for deposition and hence decelerated the UPD.

OPD occurs in potentials above thermodynamic equilibrium potentials [39, 47]. The structure and properties of the OPD deposits highly depend on various factors, including overpotential (the difference between applied and equilibrium potentials), electrolyte concentration, growth mechanism and deposit–substrate interactions [41]. Notably, diffusion-controlled nucleation is often the rate-determining step of OPD, while that of UPD is the deposit lattice incorporation into substrate [46].

### Galvanostatic (Constant Current) Deposition

**Fig. 4:**
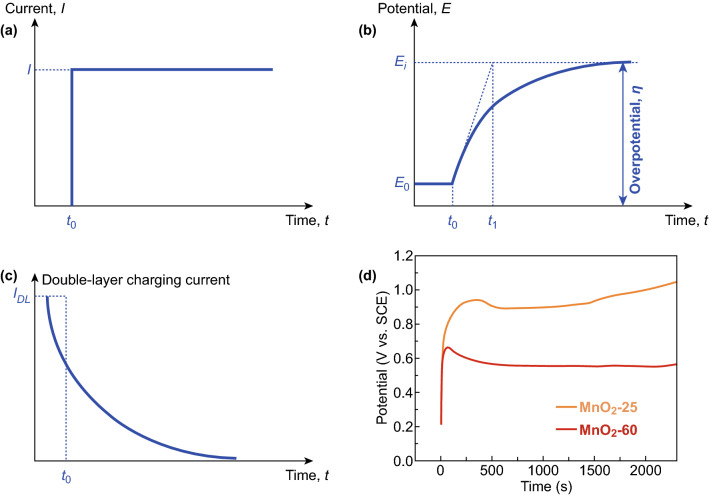
**a** Constant current applied during a galvanostatic deposition; *t*_0_ represents the moment when current is applied. **b** Potential response as a function of time in a galvanostatic deposition; *E*_0_ and *E*_i_ are the equilibrium potential and maximal potential of a working electrode during galvanostatic deposition, respectively. **c** Double-layer charging current (*I*_DL_) decays exponentially with time. **d** Time evolutions of the potential during galvanostatic depositions of MnO_2_ at temperatures of 25 °C (orange) and 60 °C (red). Adapted from Ref. [50] with permission. (Color figure online)

Galvanostatic deposition refers to electrodeposition with constant currents between the positive and negative electrodes in a two-electrode system, or between the working electrode and counter electrode in a three-electrode setup (Fig. 4a) [39]. The recorded response is the time-dependent potential of the cell (two-electrode) or the working electrode (three-electrode). The *V* − *t* curves (Fig. 4b) sometimes are called polarization curves. Unlike potentiostatic deposition that can start the moment when potentials are applied, galvanostatic deposition needs a short period to begin [48, 49]. It is because that some applied current needs to charge electrical double layers (EDLs) first. After potential reaches certain thresholds (usually equilibrium potentials plus overpotentials), electrochemical reactions occur. Therefore, the applied constant current (*I*) is contributed from two components:

2 $$I = I_{\text{DL}} + I_{\text{ct}}$$

where *I*_DL_ is capacitive current for charging EDLs and *I*_ct_ is charge transfer current for electrodeposition. *I*_DL_ rapidly approaches zero when electrodeposition starts.

*V* − *t* curves of galvanostatic deposition contain essential information on electrodeposition chemistries. Since EDL charging time is on the order of milliseconds, *V* − *t* curves collected on the timescale of minutes or hours are almost contributed from electrodeposition. For example, the *V* − *t* curves of manganese dioxide (MnO_2_) deposition processes at different temperatures qualitatively elucidate the nucleation kinetics (Fig. 4d) [50]. The increased potentials at the beginning of the electrodeposition corresponded to the nucleation of MnO_2_ as nucleation demanded more energy than its growth to surmount the activation energy barrier. Increasing temperature from 25 to 60 °C offered the additional electrodeposition energy to enable the MnO_2_ deposition at the reduced deposition potentials. The prolonged nucleation process at 25 °C results in dense manganese oxide nanosheets.

### Pulse Electrodeposition

**Fig. 5:**
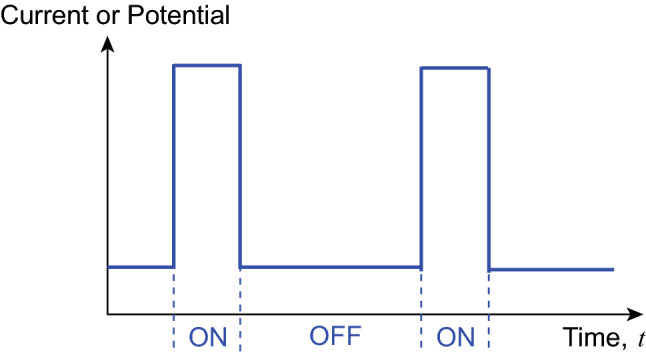
Current or potential signals applied in pulse electrodeposition

Pulse electrodeposition technique deposits materials by applying pulses of potential or current, i.e., a series of pulses with equal polarization, amplitude, and duration, separated by periodic zero current or open-circuit potentials (Fig. 5) [39, 51]. Each pulse has “on” periods when the current or potential is applied and “off” periods with no current or potential (Fig. 5) [52–54]. During the “off” periods, ions in electrolytes diffuse into electric double layers along the surfaces of the deposition substrates, which is beneficial to obtain the uniform deposition of fine-grained deposits during “on” periods [55–57].

Using pulsed electrodeposition, Yu et al. synthesized flexible graphene/polypyrrole composite films as pseudocapacitor electrodes [57]. The “off” period allowed pyrrole monomers to diffuse into the intersheet spaces of graphene and then electropolymerized into uniform polypyrrole coatings over the “on” periods. By contrast, the continuous, un-pulsed electrodeposition triggered the fast polymerization of pyrrole near the graphene sheets. Since there are no “off” periods to replenish pyrrole monomers near graphene surfaces, the lowered reactant concentration led to scattered polypyrrole particles on the graphene surface. This work highlights the suitability of pulse electrodeposition in coating uniform films onto irregularly shaped substrates.

### Electrophoretic Deposition

Electrophoretic deposition (EPD) differs from all the above-discussed techniques. First, the charge carriers in EPD are suspending, charged colloidal particles, not ions. Second, EPD involves electrostatic attractions between the particles and substrates, but no charge transfer. Third, unlike electrodeposition that demands electrolytes to conduct ions, EPD can perform in poorly conductive media, e.g., water [58, 59].

**Fig. 6:**
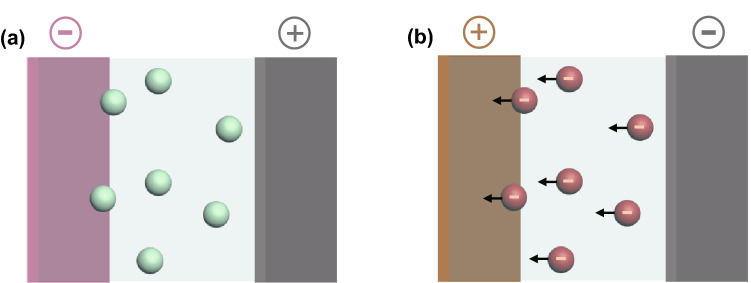
Schematic illustrations of **a** cathodic and **b** anodic electrophoretic deposition. Only ions of interest are shown for brevity

Depending on charges carried by the colloidal particles, EPD is classified into cathodic and anodic EPD. The cathodic EPD refers to the deposition of positively charged particles onto negatively charged substrates (Fig. 6a), whereas anodic EPD proceeds in a reverse manner (Fig. 6b) [58]. The structures of the deposits are tailorable by varying parameters of applied potential, particle concentration, and deposition duration [60–65]. Notably, the stoichiometry of the electrosorbed particles directly determines the stoichiometry of the deposit [59].

## Electrochemically Synthesized Carbon Materials

Carbon materials, including activated carbon, carbon fibers, carbon aerogels, carbon nanotubes, and graphene, are conventional materials in electrical double-layer capacitors [17]. Their high electrical conductivity, large surface areas, cost-effectiveness, chemical inertness, and tailorable porous structures make them suitable electrode candidates [66, 67]. The two outstanding advantages of carbon electrodes are their exceptional rate capability and ultralong lifetimes. Electrochemical approaches have been widely used to produce carbon materials [68–70]. The following sections first present the recent progress in syntheses of graphene (Sect. 3.1) and 3D carbons (Sect. 3.2), the two carbon materials that have been prepared by electrochemical technologies. Their applications in supercapacitors are highlighted separately in Sect. 3.3.

### Graphene

Electrochemical exfoliation is a facile method for synthesizing graphene. Compared with chemical exfoliation, electrochemical exfoliation avoids chemical treatments that can introduce unwanted species, simplifying product purification [66, 71]. Electrochemically exfoliated graphene often maintains more *sp*^2^-hybridized carbon networks than that made by chemical oxidation. Additionally, the graphene surface functionalization often accompanies with exfoliation [72, 73], and the heteroatom doping level is highly controllable. Another strength of electrochemical exfoliation is its high efficiency. It only needs minutes or hours, depending on applied potentials, electrolyte compositions, and graphite sources, to produce grams of graphene sheets in laboratories [74, 75].

According to the potential polarity, electrochemical exfoliation is classified into (1) anodic exfoliations performed in aqueous electrolytes containing inorganic salts [76], mineral acids [77, 78], ionic liquids [79], or their mixtures [67, 80]; and (2) cathodic exfoliations in organic electrolytes having lithium or alkylammonium salts [21, 67, 81–83].

#### Anodic Exfoliation

**Fig. 7:**
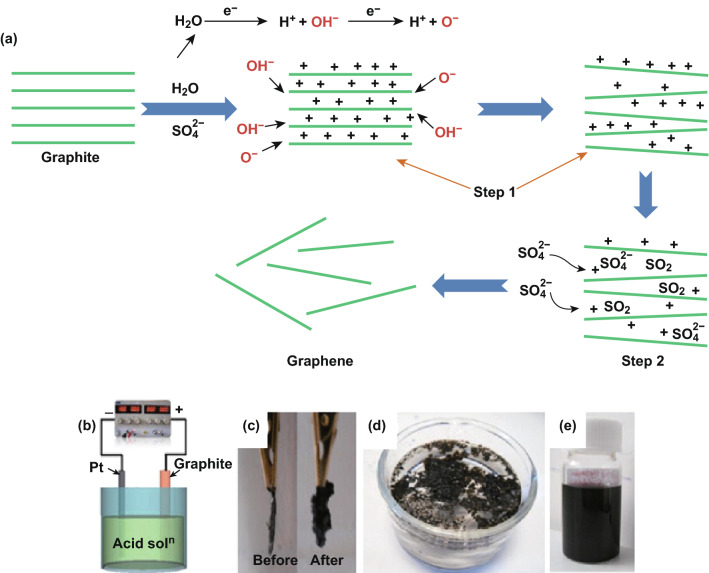
**a** Schemes of the microscopic processes of graphite exfoliation in H_2_SO_4_ aqueous electrolytes. Step 1: edge opening by waterborne radicals; Step 2: SO_4_^2−^ intercalation and exfoliation. **b** Experimental setup for the graphite exfoliation. **c** Photographs of the exfoliated electrodes before and after exfoliation. **d** Exfoliated graphene floating on an electrolyte. **e** Dispersed graphene sheets in dimethylformamide solution. Adapted from Ref. [84] with permission

Anodic exfoliation separates graphite into graphene by anion intercalation [73, 76]. It is the most used electrochemical approach for graphene production due to its high exfoliation efficiency. With inorganic acids (e.g., H_2_SO_4_ [78, 84], HNO_3_ [85, 86], and H_3_PO_4_ [87]) or salts (e.g., KNO_3_ [88] and (NH_4_)_2_SO_4_ [89]) as supporting electrolytes, a high exfoliation potential (e.g., 3–10 V) can generate single-layer or multilayer graphene sheets from graphite. For example, Parvez et al. exfoliated graphite foils in sulfuric acid aqueous solutions with concentrations of 0.1, 1, and 5 M [84]. The synthesis procedures involved multiple steps (Fig. 7a). First, a high potential of 10 V was applied across a graphite positive electrode and a Pt negative electrode (Fig. 7b), splitting water into hydroxyl (OH·) and oxygen radicals (O·). These radicals preferentially oxidized the boundaries and defects of graphite, opening its edges. Second, driven by the applied electrical field, sulfate ions (SO_4_^2−^), together with water molecules, intercalated into graphite layers through the open edges and expanded graphite layers. Meanwhile, oxygen gas evolution in between graphite layers further torn apart graphite sheets and dispersed exfoliated graphene layers into electrolytes (Fig. 7c–e). Water in the electrolytes was critical for electrochemically exfoliation, as it both generated OH· and O· (the exfoliation initiators) and served as an intercalant. The exfoliation efficiency in the 0.1 M H_2_SO_4_ electrolyte reached the highest, since the 5 M H_2_SO_4_ electrolyte overexfoliated graphite into graphitic particles, while the 0.1 M H_2_SO_4_ electrolyte led to incomplete exfoliation due to insufficient sulfate ions. The obtained graphene sheets in 0.1 M H_2_SO_4_ had a high yield of > 80%, less than three layers, a high C/O ratio of 12.3, and good electrical conductivity (sheet resistance of ~ 4.8 kΩ/□), all of which are comparable to those of high-quality graphene sheets synthesized by chemical vapor deposition.

Changing the water content of non-aqueous electrolytes can yield graphene-based materials with different surface areas and morphologies. Specifically, Lu et al. found that the morphologies of the exfoliation products in 1-methyl-3-butylimidazolium tetrafluoroborate, an ionic liquid (IL), depended on the water-to-IL ratio [79]. Identical to electrochemical exfoliations in aqueous solutions, water in the IL produced OH· and O· to drive the exfoliation process, while subsequent intercalation of BF_4_^−^ promoted complete exfoliation. Decreasing the water/IL ratio favored BF_4_^−^ intercalation that significantly expanded graphite into graphene nanoribbons. Increasing the water/IL ratio increased the populations of OH· and O·, which substantially oxidized and broke graphite into hydroxylated carbon particles. In this case, the oxygen-containing radicals acted as electrochemical “scissors” that cut the graphite plates into nanoribbons or nanoparticles.

Graphite exfoliation in aqueous solutions often yields oxygenated graphene with reduced electrical conductivity, a property unfavorable for rapid charge storage [36, 80, 90–94]. To circumvent this shortcoming, a variety of additives, including reducing agents [95] and oxygen radical scavengers [76], have been introduced into electrolytes to prevent overoxidation of exfoliated graphene. For example, Yang et al. demonstrated that (2,2,6,6-tetramethylpiperidin-1-yl)oxyl (TEMPO), ascorbic acid, and sodium borohydride could consume radicals. This characteristic kept oxygen content at low levels (3.8 atom% O of TEMPO-added exfoliated graphene vs. 11 atom% O of TEMPO-free exfoliated graphene) [95]. Besides, Ejigu et al. reported that transition metal ions (e.g., Co^2+^, Ni^2+^, Fe^3+^, Mn^2+^, Ru^3+^, Ir^3+^, and V^3+^) as electrolyte additives were conducive to acquiring high-quality graphene because they scavenged oxygen radicals [76]. Among all these ions, Co^2+^ was the most promising one because it converted to an oxygen evolution reaction catalyst. During anodic exfoliation, Co^2+^ was first oxidized to Co^4+^, an active species for oxygen evolution. The Co^4+^ adsorbed on graphite facilitated oxygen evolution from water, bypassing the formation of oxygen radicals that oxidized exfoliated graphene. The lowest oxygen content was 2.6%.

#### Cathodic Exfoliation

**Fig. 8:**
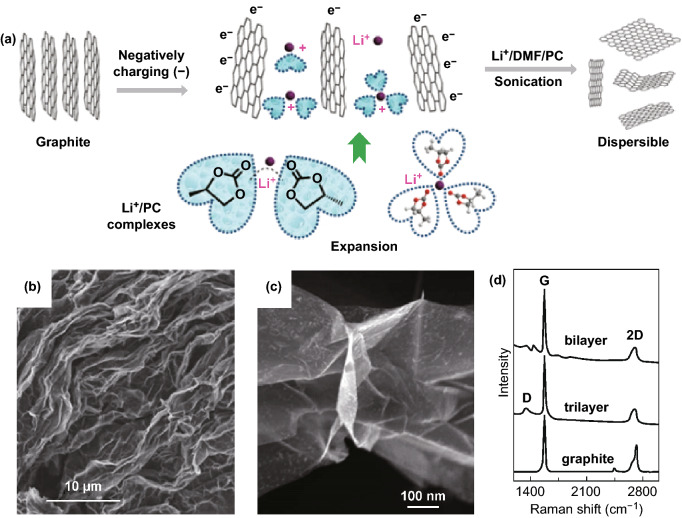
**a** Schematic illustration of cathodic exfoliation of graphite by intercalation of Li^+^ complexes. **b**, **c** SEM images of the cathodically exfoliated graphene plates. **d** Raman spectra of the cathodically exfoliated graphene with bilayers and trilayers, in comparison with that of pristine graphite. Adapted from Ref. [21] with permission

Cathodic exfoliation, which produces graphene by applying negative biases to graphite, is a method free of oxidation concerns. Though this technique is not as developed as anodic exfoliation, it has successes in graphite exfoliation in organic-based electrolytes [21, 67, 81, 83] and molten salts [82]. For example, Wang et al. deployed a cathodic exfoliation method that acquired highly conductive, less than five layers of graphene nanosheets from graphite with yields of > 70% [21]. At a high cathodic potential of − 15 ± 5 V, Li^+^–propylene carbonate (PC, a solvent) complexes intercalated into graphite and expanded graphite layers. Subsequently, the reduction of PC molecules liberated gas bubbles in between graphite layers, eventually exfoliating graphite into graphene sheets (Fig. 8a–c). Because the exfoliation involved no oxidation, the resultant graphene contained little defects, as evidenced by the small *I*_D_/*I*_G_ ratio of 0.1 revealed by Raman spectroscopy (Fig. 8d).

In addition to liquid electrolytes, quasi-solid molten salts were potent electrolytes for graphite exfoliation. For instance, Huang et al. successfully exfoliated graphite in molten LiOH with a high cathodic current of 15 A [82]. This process was based on intercalation, expansion, and micro-explosion. First, Li^+^ ions in the molten LiOH intercalated into graphite layers and widened the interlayer gap of graphite, forming graphite intercalation compound (Li_*x*_C_*y*_). Afterward, the Li^+^-intercalated graphite was soaked in water. Li_*x*_C_*y*_ and metallic Li reacted violently with water (micro-explosion), creating hydrogen gas bubbles that further exfoliated graphite into graphene. The conversion efficiency was ~ 80%.

### Three-Dimensional Carbons

As supercapacitor electrodes, three-dimensional (3D) carbon materials have advantages over other conventional carbon powders (e.g., activated carbon) and graphene. First, their self-standing nature requires no binder blending for preparing electrodes, which eases fabrication and reduces the negative impact of the binders on electron transport. Second, their tailorable structures offer opportunities to achieve both high surface areas and hierarchical porous networks known to facilitate ion diffusion [4, 96–102].

**Fig. 9:**
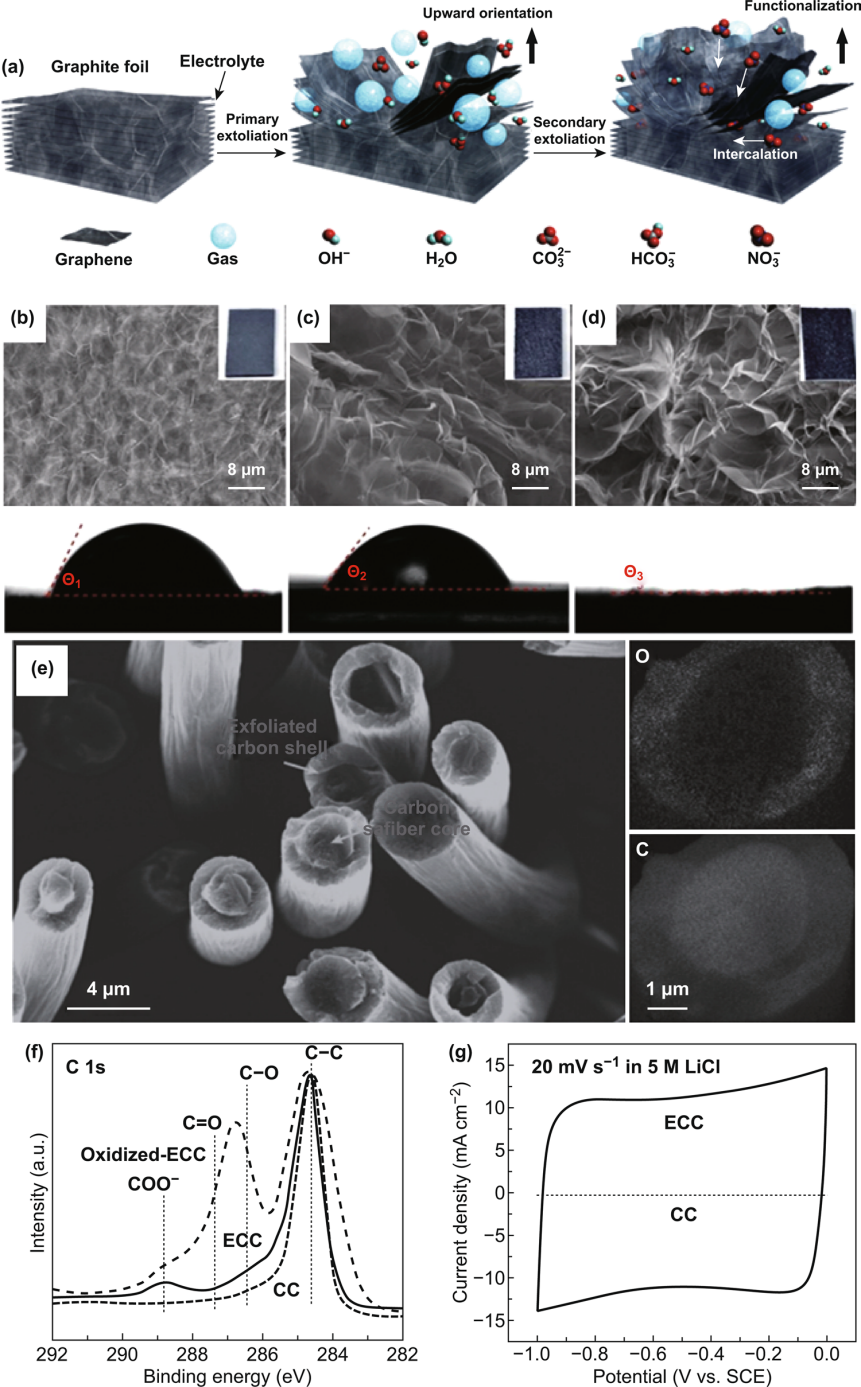
**a** Schemes of the two-step partial exfoliation of graphite foil. **b**–**d** SEM images and contact angles of **b** graphite foil, **c** graphite foil after primary exfoliation, and **d** graphite foil after secondary exfoliation. Insets: photographs of graphite foil at different treatment stages. **e** SEM image and EDS element mappings (C and O) of the cross sections of exfoliated carbon cloth fibers. **f** XPS C 1*s* spectra of pristine carbon cloth (CC) and exfoliated carbon cloth (ECC). **g** CV curves of CC and ECC. Adapted from **a**–**d** Ref. [88] and **e**–**g** Ref. [37] with permission

Electrochemical partial exfoliation of graphitic materials is the most common synthesis method of electrochemically synthesizing 3D carbon materials. As indicated by its name, electrochemical partial exfoliation only partially exfoliates graphitic precursors, e.g., carbon fibers [37, 85, 97, 99], graphene aerogels [99], and graphite foils [88, 103, 104], leading to graphene sheets anchored on the exposed surfaces. For example, Song et al. demonstrated a two-step electrochemical partial exfoliation method to prepare oxygen-functionalized, partially exfoliated graphite foils (Fig. 9a) [88]. The authors first scanned a piece of graphite foil (EG in Fig. 9b) in aqueous K_2_CO_3_ electrolytes by cyclic voltammetry. This step partially exfoliated the graphite layers on and near the outer surface of EG through vigorous gas evolution from water splitting (Fig. 9c). The secondary exfoliation process intercalated NO_3_^−^ into EG through the open edges and defects formed during the first step, forming graphite intercalation compounds (C-NO_3_). These compounds, when placed in water, hydrolyzed and released oxygen gas. The gas evolution further exfoliated and oxygenated the top layers of EG (Fig. 9d). The introduced oxygen functionalities rendered the foil superhydrophilicity as reflected from the zero contact angle (Fig. 9d inset). The potential applied in the second step controlled the degrees of the exfoliation and oxygenation. The functionalized, exfoliated EG possessed a 3D network consisting of oxygenated graphene sheets integrated onto graphite foil. The seamless integration between the top layer and the graphite bottom ensured fast electron conduction pathways. Besides improving electrolyte wettability, the oxygen moieties served as anchoring sites for depositing guest materials for polyaniline [27], polypyrrole [105], manganese oxides [86], vanadium oxides [37], iron oxides [35, 106], nickel–cobalt double hydroxides [106], and molybdenum-based materials [28].

In addition to graphite, carbon fibers can also be partially electroexfoliated. For example, Wang et al. synthesized electrochemically activated carbon fiber cloth electrodes by applying a voltage of 3 V in HNO_3_/H_2_SO_4_ mixed aqueous electrolytes [85]. The high voltage, together with the highly oxidative and corrosive acids, roughened the carbon fiber surfaces and introduced functionalities of C–OH, —C=O, and —COOH. This activated carbon cloth electrode exhibited a high areal capacitance of 756 mF cm^−2^ at 6 mA cm^−2^. Song et al. demonstrated an acid-free method to exfoliate carbon fiber cloth partially [37]. First, NO_3_^−^ anions intercalated into carbon fibers, exfoliating and oxidizing the outer surface (Fig. 9e). To recover the electrical conductivity of carbon fibers, the researchers immersed the oxidized carbon cloth in a 0.1% hydrazine hydrate aqueous solution to reduce the oxygen content. This reduction process removed most of the oxygen functionalities (Fig. 9f) and resulted in enhanced capacitive performance (Fig. 9g). The partially exfoliated carbon cloth electrode exhibited a high areal capacitance of ~ 500 mF cm^−2^ at 20 mA cm^−2^.

### Application for Supercapacitors

Owing to the enhanced surface areas and high electrical conductivity, electrochemically exfoliated graphene-based materials or activated carbon fibers have functioned as either electrode materials or current collectors in supercapacitors. Liu et al. fabricated an in-plane micro-supercapacitor through directly printing electrochemically exfoliated graphene on patterned microelectrodes. This micro-supercapacitor delivered an areal capacitance of 800 μF cm^−2^ at 1 mV s^−1^ [107]. Wu et al. used electrochemically exfoliated graphene to prepare graphene paper and 3D graphene foams as supercapacitor electrodes [75]. The specific capacitance of the 3D graphene electrode reached 113.2 F g^−1^ and 58.9 F g^−1^ at 0.5 A g^−1^ in 6 M KOH aqueous and 1 M triethylmethylammonium tetrafluoroborate acetonitrile (TEMABF_4_/AN), respectively.

Besides reporting the outstanding electrochemical performance, some researchers devoted to revealing the interplays between exfoliation conditions and electrochemical properties. Ambrosi et al. compared the capacitive performances of electrochemically exfoliated graphene synthesized in different aqueous electrolytes, i.e., 0.5 M H_2_SO_4_, Na_2_SO_4_, and LiClO_4_ [71]. The results showed that the graphene prepared in H_2_SO_4_ and Na_2_SO_4_ exhibited relatively high specific capacitance of 78 and 106 F g^−1^, respectively. LiClO_4_ introduced a large amount of oxygen functional groups on the exfoliated graphene, which could be anchoring sites for growing other materials to form graphene-based composites. Notably, the specific capacitances of the electrochemically exfoliated graphene often fall in the range of 50–100 F g^−1^, which are slightly lower than those of graphene obtained through chemical oxidation. The underlying reason was revealed by Xia et al., who studied the different graphite exfoliation routes via chemical oxidation and electrochemical exfoliation [66]. The authors discovered that the surface area of the electrochemically exfoliated graphite was only 6.6 m^2^ g^−1^, which was three orders of magnitude lower than that of the theoretical value (2600 m^2^ g^−1^) of a graphene monolayer achieved by chemical exfoliation. Therefore, electrochemical exfoliation is challenging to fully separate graphite into monolayer graphene, resulting in the relatively low gravimetric capacitance due to the small surface area (Table 1). This limitation has motivated the introduction of pseudocapacitive functional groups to boost capacitance [85, 99, 101].

**Table 1:** Synthesis conditions and specific capacitance of electrochemically exfoliated graphene-based electrodes

Material^a^	Method	Exfoliation electrolyte^b^	Specific capacitance	Current density/scan rate
Exfoliated carbon cloth [85]	Anodic exfoliation	HNO_3_/H_2_SO_4_	756 mF cm^−2^	6 mA cm^−2^
Exfoliated carbon fibers [102]	Cathodic exfoliation	0.1 M TMAClO_4_/NMP	64.5 mF cm^−2^	10 mV s^−1^
Partial exfoliation of HOPG [104]	Anodic exfoliation	1 M H_2_SO_4_	750 mF cm^−2^	20 mV s^−1^
Exfoliated carbon paper [86]	Anodic exfoliation	0.5 M KOH	400 mF cm^−2^	1 mA cm^−2^
Trilayer graphite foil [101]	Anodic exfoliation	0.5 M K_2_CO_3_/0.5 M KNO_3_/3 M KCl	820 mF cm^−2^	5 mA cm^−2^
Exfoliated carbon cloth [97]	Anodic exfoliation	2 M H_2_SO_4_	12 mF cm^−2^	1 mA cm^−2^
Exfoliated graphitic paper [99]	Cathodic and anodic exfoliation	1 M LiClO_4_/propylene carbonate	108.8 mF cm^−2^	0.5 mA cm^−2^
Exfoliated 3D printed graphene aerogel [99]	Cathodic and anodic exfoliation	1 M LiClO_4_/propylene carbonate	101.7 F g^−1^	10 A g^−1^
Vertically oriented graphene nanosheets [98]	Anodic exfoliation	0.5 M H_2_SO_4_	3.9 F cm^−3^	7.5 mA cm^−3^
Exfoliated graphite foils [88]	Anodic exfoliation	0.5 M K_2_CO_3_/1 M KNO_3_ in PBS (pH = 6.7)	60 mF cm^−2^	0.23 mA cm^−2^
Exfoliated carbon cloth [37]	Anodic exfoliation	0.5 M KNO_3_	560 mF cm^−2^	2 mA cm^−2^
Graphene [108]	Anodic exfoliation	0.1 M NaI	50 F g^−1^	0.1 A g^−1^
Graphene [107]	Anodic exfoliation	0.1 M H_2_SO_4_	5.4 mF cm^−2^	1 mV s^−1^
Graphene [92]	Anodic exfoliation	0.1 M (NH_4_)_2_SO_4_	56.6 F g^−1^	1 A g^−1^
Graphene [95]	Anodic exfoliation	0.05 M (NH_4_)_2_SO_4_ + TEMPO + H_2_O	11.5 mF cm^−2^	2 mV s^−1^
Graphene [89]	Anodic exfoliation	0.1 M (NH_4_)_2_SO_4_	11.3 mF cm^−2^	1 mV s^−1^
S-doped graphene [109]	Anodic exfoliation	Na_2_S_2_O_3_ + H_2_SO_4_	320 F g^−1^	3 A g^−1^
Graphene film [110]	Anodic exfoliation	0.15 M Na_2_SO_4_ + 0.01 M sodium dodecyl sulfate	900 μF cm^−2^	10 mV s^−1^
Graphene aerogel [111]	Cathodic/Anodic exfoliation	0.2 M H_2_SO_4_ + KOH	325 F g^−1^	1 A g^−1^
3D graphene [75]	Anodic exfoliation	10 M H_2_SO_4_	113.2 F g^−1^	0.5 A g^−1^
3D N-doped graphene [112]	Anodic exfoliation	CH_3_COOH/H_2_SO_4_ (V/V = 4:6)	170.5 F g^−1^	10 mV s^−1^
Acid modified graphene [113]	Anodic exfoliation	9-anthracene carboxylic acid (1 mg mL^−1^)	577 F g^−1^	7 A g^−1^
Graphene film [114]	Electrophoretic deposition	Graphene oxide suspension (0.6 mg mL^−1^)	156 F g^−1^	0.15 A g^−1^
P-doped graphene [115]	Anodic exfoliation	H_3_PO_4_	290 F g^−1^	1 A g^−1^
Graphene [71]	Anodic exfoliation	0.5 M Na_2_SO_4_	106 F g^−1^	0.1 A g^−1^
Graphene [71]	Anodic exfoliation	0.5 M LiClO_4_	78 F g^−1^	0.1 A g^−1^
Graphene [71]	Anodic exfoliation	0.5 M H_2_SO_4_	21 F g^−1^	0.1 A g^−1^

^a^TMAClO_4_: tetramethylammonium perchlorate; NMP: *N*-methyl pyrrolidone; PBS: phosphate-buffered saline; TEMPO: (2,2,6,6-tetramethylpiperidin-1-yl)oxyl

^b^Unless specifically stated, solutions are aqueous-based

Compared with fully exfoliated graphene sheets, partial exfoliated graphite and carbon electrodes have the main advantage that the whole electrodes remain structurally and electrically connected, enabling them to function as current collectors for loading pseudocapacitive materials. The resultant composite materials possess both high capacitance from the incorporated pseudocapacitive materials and the excellent rate capability characteristic of carbon-based materials. In this regard, the mass loadings of the pseudocapacitive materials must be meticulously tuned to ensure the highest specific capacitance without significantly compromising the rate capability.

## Conducting Polymers

Conducting polymers, or conjugated polymers, are organic polymers that conduct electricity in their electron conjugated networks in the polymer backbones [116–118]. Polyaniline (PANI) [119–122], polypyrrole (PPy) [105, 123], polythiophene (PTh) [124, 125], and poly(3,4-ethylene dioxythiophene) (PEDOT) [126–129] are common supercapacitor electrode materials. In terms of electrochemical synthesis, electrochemical polymerization of monomers is typical to prepare these conducting polymers. It grows conducting polymers onto electrically conductive substrates (current collectors), eliminating the need for blending powdered materials with binders and conductive additives when preparing electrodes. Potentiostatic deposition [130], galvanostatic deposition [131, 132], and cyclic voltammetry [105] are synthesis techniques of conducting polymers. The thicknesses and mass loadings of conducting polymers are controllable by tuning deposition duration. The compositions of electrolytes mainly influence their electrical conductivity. This section summarizes the mechanisms of electrochemical polymerization, recent progress of the electrochemically synthesized conducting polymers, and their electrochemical performance as supercapacitor electrodes.

### Mechanism of Electrochemical Polymerization of Conducting Polymer Materials

Electrochemical technologies are time- and cost-efficient in preparing conducting polymers. Electrochemical polymerization begins with oxidizing monomers possessing five-membered aromatic heterocycles (e.g., pyrrole or thiophene) [133, 134] or cyclic aromatic amines (e.g., aniline) [116]. The oxidation involves generating and dimerizing radical cations, followed by polymer chain growth. Electrochemical polymerization initiates polymer growth on the surfaces of conductive substrates.

**Fig. 10:**
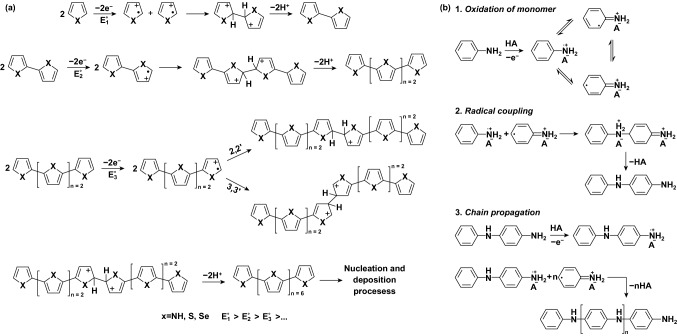
**a** Electrochemical polymerization mechanism of pyrrole. Adapted from Ref. [133] with permission. **b** Electrochemical polymerization mechanism of polyaniline. Adapted from Ref. [135] with permission

Diaz proposed the widely accepted electrochemical polymerization mechanism of five-membered aromatic monomers in 1983 (Fig. 10a) [133]. Taking polypyrrole (PPy) as an example, pyrrole (Py) monomers are first oxidized to radical cations under an anodic potential. Subsequently, the radical cations dimerize through radical–radical coupling reactions at *α*-positions and deprotonated into neutral dimers. The as-formed dimers further combine and eventually extend to PPy.

The polymerization of another conducting polymer, polyaniline, follows a similar path as that of PPy (Fig. 10b) [135]. Its monomers are first oxidized to radical cations, then coupled, and deprotonated to dimers. Unlike PPy, whose chain propagation is driven by continuous dimer combination, aniline dimers undergo further oxidation and couple with one aniline radical cation at a time. The coupling extends to polyaniline. For both cases, anions (or counterions in general) will dope into the as-formed conducting polymers to balance the charges carried by the positively charged sites on the polymer backbones. Note that the concept of doping in the context of conducting polymers is fundamentally different from that in conventional solid-state semiconductors. Doping semiconductors means to incorporate dopants into the crystal lattices of the host materials [136].

This counterion doping process maintains the electroneutrality of conducting polymer and affects the electrical conductivity of conducting polymers [137]:

3 $${\text{Pol}}^{ *} + n{\text{A}}^{ - } + m{\text{S}} \leftrightarrow {\text{Pol}}^{{{\text{n}} + }} {\text{A}}^{ - } {\text{S}} + ne^{ - }$$

where Pol* is the positively charged sites in a conducting polymer, A^−^ stands for counterion, and S represents a solvent molecule. When electrons enter in conducting polymers, doping of A^−^ and concurrently co-insertion of solvent molecules lead to volumetric expansion of the host polymers. Conversely, electron extraction causes de-doping of A^−^ and de-solvation of conducting polymers, resulting in volumetric contraction. The irreversible volumetric deformation is a typical culprit for the structural instability that causes unsatisfactory cycling stability of conducting polymers [138, 139].

### Electrochemically Synthesized Conducting Polymers for Supercapacitors

#### Films

Conformal films are the most common morphologies of electrochemically polymerized conducting polymer electrodes [97, 140, 141]. Parameters associated with electropolymerization have profound influences on the chemical compositions, morphologies, and electrochemical properties of the deposited polymer films [130, 142].

The surface properties of the substrates influence the adhesion strength and chemical compositions of the polymer films. For example, Feng et al. electrochemically deposited a thin PPy layer on oxygen-functionalized carbon cloth (FCC) [97]. Compared with PPy deposited on pristine carbon cloth (CC), PPy/FCC exhibited enhanced dopant concentrations and electrical conductivity, because the oxygen functional groups on FCC could dope into PPy and reinforced the adhesion of PPy onto FCC. Consequently, PPy/FCC displayed an areal capacitance of 341 mF cm^−2^ at 1 mA cm^−2^, about 40 mF cm^−2^ higher than that of PPy/CC at the same current density.

**Fig. 11:**
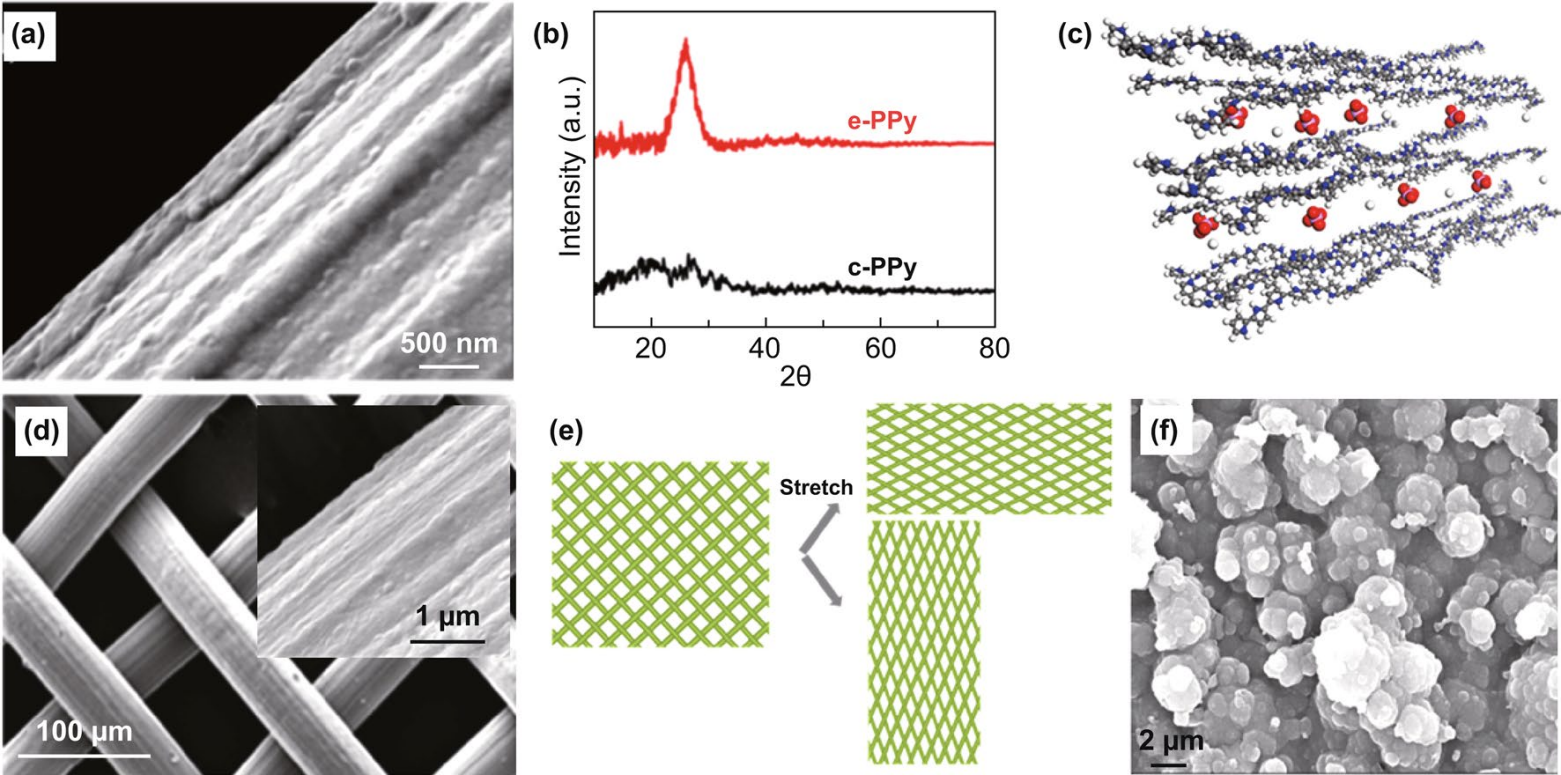
**a** SEM image of electrodeposited PPy film on an oxygen-functionalized carbon fiber. **b** XRD patterns of electrochemically and chemically deposited PPy. **c** A possible molecular structure of electrodeposited PPy. **d** PPy film electrochemically deposited on a flexible stainless steel mesh. **e** Schematic illustration of the stretched mesh structure. **f** Cauliflower-like PPy film. Adapted from **a**–**c** Ref. [143], **d**, **e** Ref. [132], **f** Ref. [105] with permission

Electrochemically polymerized conducting polymer films have different molecular structures from those prepared by chemical polymerization. Huang et al. reported that PPy film deposited via galvanostatic electrodeposition exhibited higher molecular order than that made by chemical oxidation (Fig. 11a) [143]. During electrochemical polymerization, the α–α coupled PPy chains stacked layer by layer with an interlayer spacing of 3.45 Å, as evident from the pronounced X-ray diffraction peak (Fig. 11b, c). This layered molecular structure facilitated ion transport within the electrode and induced a homogeneous stress distribution in the polymer films, both of which improved the cycling stability of PPy.

Coating conducting polymer films onto flexible substrates is a strategy of making flexible electrodes for wearable supercapacitors. Huang et al. demonstrated a stretchable stainless steel mesh as an electrically conductive substrate to endow the deposited PPy film excellent stretchability (Fig. 11d) [132]. The PPy-coated stainless steel mesh delivered a specific capacitance of 170 F g^−1^ at 0.5 A g^−1^, and the capacitance augmented to 214 F g^−1^ when the electrode was applied a 20% strain. The strain improved the contact between PPy and stainless steel and reduced the contact resistance, which augmented the specific capacitance (Fig. 11e).

It should be noted that thin films are usually obtained at the early stage of electrode polymerization, and prolonging deposition time may modify the film morphology due to overgrowth. For instance, Song et al. observed that instead of thin films, PPy cauliflowers formed (Fig. 11f) after 10 cycles scanning from 0 to 0.8 V vs. SCE at 50 mV s^−1^ [105].

**Fig. 12:**
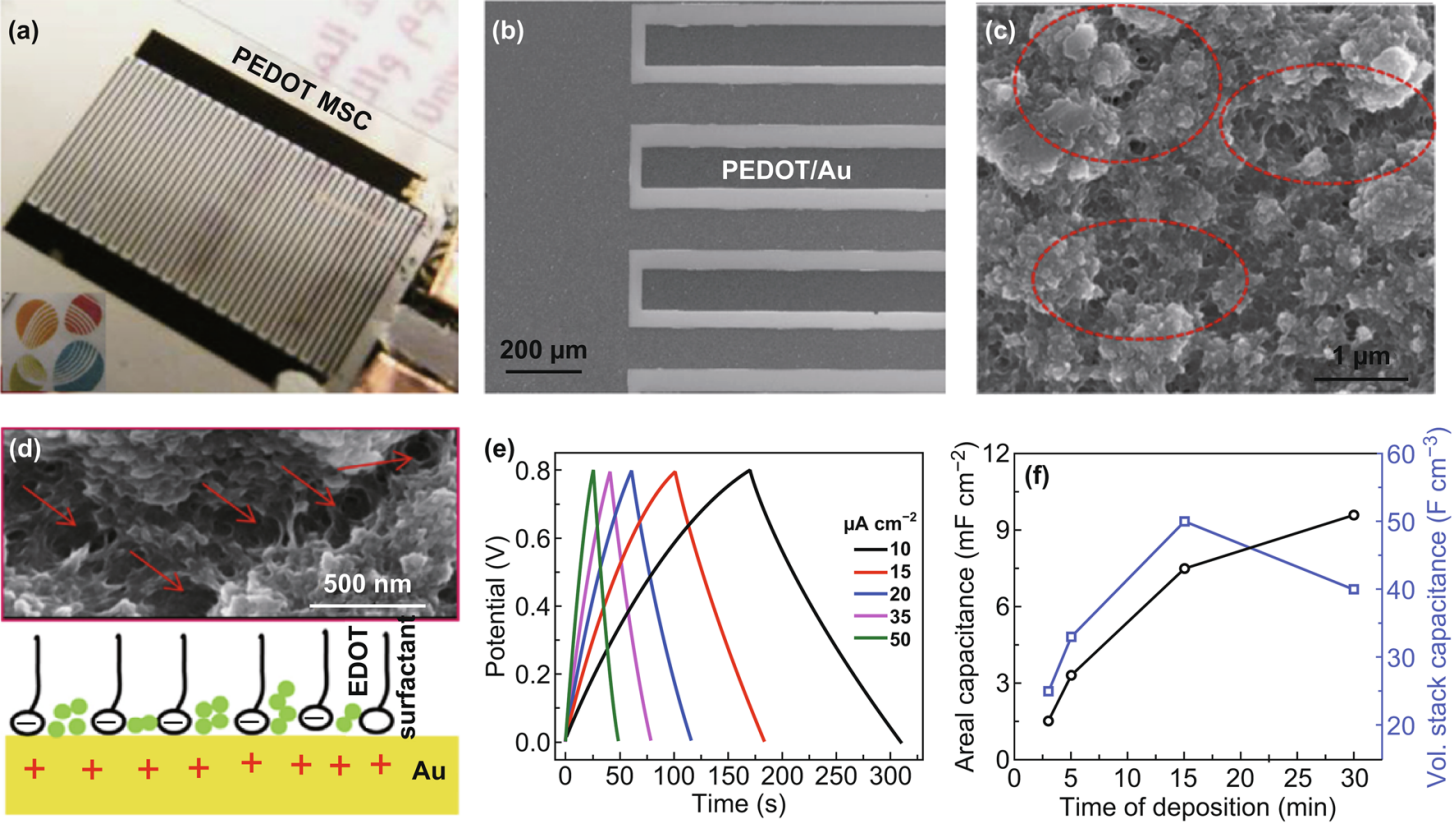
**a** Photograph and **b** SEM image of PEDOT-coated interdigitated electrode. **c** Magnified view SEM image of the electrodeposited PEDOT film. The red circles highlight the cracks formed by soft templates. **d** Scheme illustrating the surfactant-induced crack formation. **e** Galvanostatic charge–discharge profiles of a symmetric micro-supercapacitor consisting of two PEDOT-coated interdigitated electrodes. Electrolyte: 1 M H_2_SO_4_ aqueous solutions. **f** Areal and volumetric capacitances of the symmetric micro-supercapacitor as a function of polymer deposition time. Adapted from Ref. [144] with permission

Soft templates (e.g., surfactant) can introduce porosity in electrodeposited conducting polymer films. Kurra et al. used a potentiostatic method to deposit a thin layer of PEDOT on an Au-coated, interdigitated electrode (Fig. 12a, b) [144]. Sodium dodecyl sulfate, an anionic surfactant, was used to increase the solubility of 3,4-ethylenedioxythiophene (EDOT) in water and thus decreased the polymerization potential of EDOT. The surfactant molecules also served as soft templates that created cracks in the PEDOT film (Fig. 12c, d). These cracks provided electrolyte ion percolation pathways and benefited rate capability at high frequencies. A symmetric micro-supercapacitor consisting of two identical interdigitated electrodes displayed a typical capacitive behavior as reflected from the plateau-free galvanostatic charge–discharge profiles (Fig. 12e). This micro-supercapacitor exhibited a positive trend between its areal capacitance and the electropolymerization time, but the volumetric capacitance peaked after 15 min polymerization (Fig. 12f). The drop in the volumetric capacitance was attributed to the increased PEDOT thickness that impeded ion diffusion.

#### Nanowires and Nanorods

One-dimensional (1D) conducting polymers, such as polyaniline nanofibers [145–147] and polypyrrole nanorods [148–150], are popular morphologies of pseudocapacitor electrodes. Their merits include the wide-open interfiber space that facilitates electrolyte infiltration and ion diffusion, as well as minimizes dead volumes (materials that are unusable for charge storage).

**Fig. 13:**
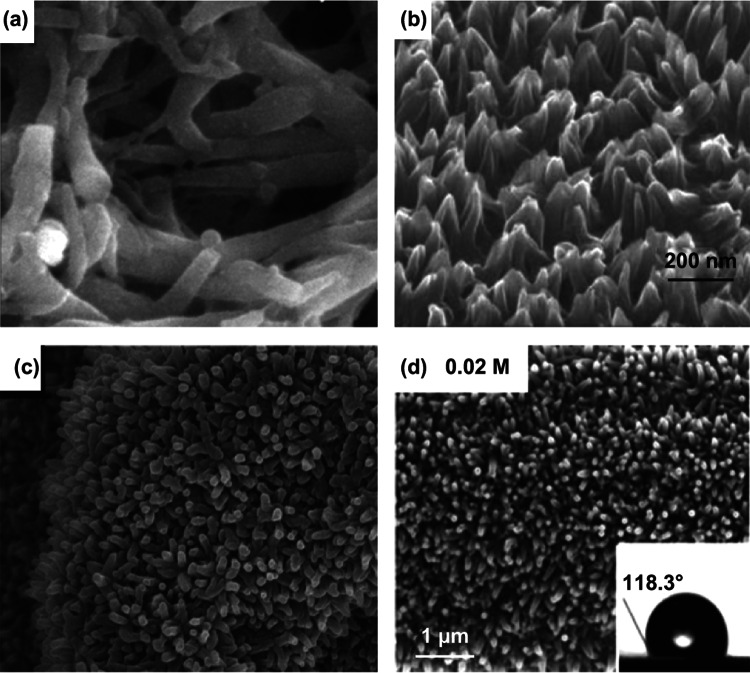
Electrochemically deposited 1D polymer structures. **a** PANI nanofibers on carbon cloth; **b** PANI nanorod arrays on Au plates; **c** PANI nanorod arrays on carbon nanotube paper; **d** PPy nanorod arrays on carbon cloth. Adapted from **a** Ref. [25], **b** Ref. [153], **c** Ref. [145], **d** Ref. [149] with permission

In the absence of any structure-directing agents, polyaniline preferentially forms randomly intertwined nanofibers [151]. Liu et al. electrodeposited polyaniline (PANI) nanowires on carbon cloth using cyclic voltammetry within a potential window between − 0.2 and 0.8 V in aqueous electrolytes containing 0.1 M aniline and 1 M H_2_SO_4_ [25]. PANI nanowires were uniformly grown on carbon cloth fibers (Fig. 13a). To address the intrinsic cycling instability of PANI, the researchers conformally coated the deposited PANI nanowires with 5-nm-thick carbonaceous shells by hydrothermally decomposing glucose. The coated PANI electrode exhibited a high theoretical areal capacitance of 787.4 mF cm^−2^ (estimated by the Trasatti method) and excellent cycling stability of ~ 95% after 10,000 charge–discharge cycles. SEM revealed that the carbonaceous shell mitigated the volumetric deformation-induced structural pulverization of PANI.

Confining the growth of conducting polymers from current collector surfaces is a prerequisite to obtaining binder-free supercapacitor electrodes. To suppress electropolymerization of monomers in bulk electrolytes, small current, low potential, and dilute monomer solutions are preferred. Once polymer nucleates on substrate surfaces, they minimize the energy barrier for the subsequent growth of conducting polymer nanostructures [152, 153]. For example, PANI nanorod arrays were grown on a gold plate using a galvanostatic method with a small current density of 0.01 mA cm^−2^ (Fig. 13b) [153]. The formation mechanism followed the nucleation-initiated growth process. The electrodeposited PANI nanorod array electrode exhibited a high specific capacitance of 950 F g^−1^ at 1 A g^−1^. Following the same protocol, PANI nanorod arrays were grown on other conductive substrates, such as carbon nanotubes (Fig. 13c) [145], exfoliated graphene sheets [154], as well as graphene papers [155, 156]. The generality of substrates indicated that the nucleation growth process is independent of substrate properties.

In addition to PANI, electrochemical technology also produces polypyrrole (PPy) nanorod arrays on conductive substrates. Huang et al. fabricated PPy nanorod arrays via a one-step galvanostatic deposition at 1 mA cm^−2^ with *p*-toluenesulfonate acid (TsOH) as a soft template (Fig. 13d) [149]. The TsOH anions prevented the as-formed PPy oligomers from growing in random directions, promoting the growth of PPy nanorods on carbon cloth. Significantly, the PPy nanorods exhibited capacitance of 699 F g^−1^ at 1 A g^−1^. When the current density increased from 1 to 20 A g^−1^, 81.5% capacitance retained, indicating its excellent rate capability.

**Fig. 14:**
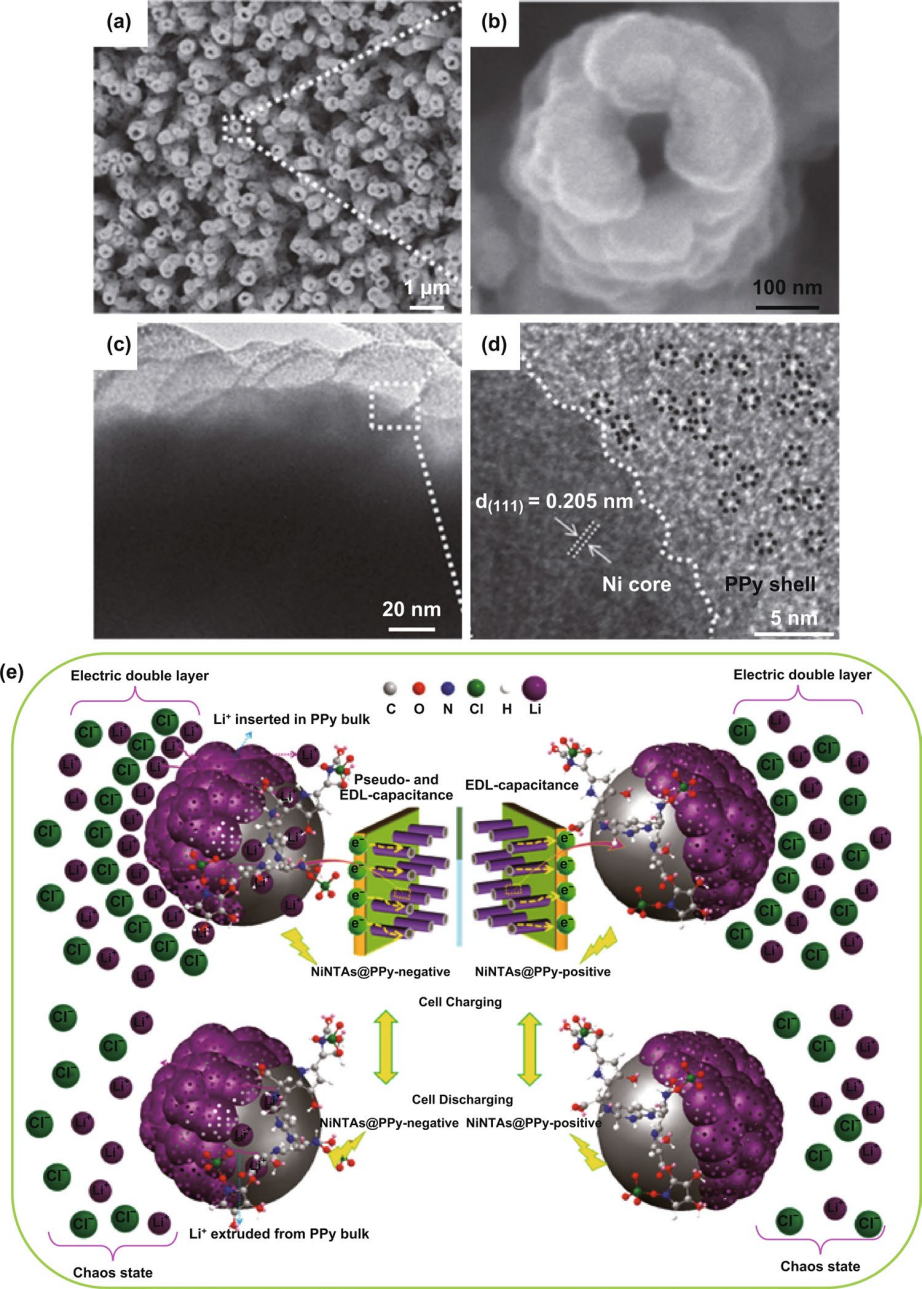
**a**, **b** SEM and **c**, **d** TEM images of NiNTAs@PPy. The black dotted circles in **d** highlight micropores in PPy. **e** Schemes showing the charge storage mechanism of NiNTAs@PPy in supercapacitors during (top) charging and (bottom) discharging. Adapted from Ref. [157] with permission

The use of hard templates enables the growth of sophisticated 1D nanostructures, such as nanotubes. Using nickel nanotube array (NiNTA) hard templates, Chen et al. made perchlorate-doped PPy nanotubes (Fig. 14) [157]. First, Ni nanoparticles were dispersed on ZnO nanorod arrays to form ZnO@NiNRAs, followed by dissolving the ZnO templates to produce NiNTAs. Second, the electropolymerization of PPy on NiNTAs generated NiNTAs@PPy (Fig. 14a, b). High-resolution TEM showed abundant mesopores throughout the PPy layer (Fig. 14c, d). Possibly, the mesopores formed during the polymerization process when the anions were inserted into PPy, and the cations were extracted. The highly porous hollow nanotube arrays in NiNTAs@PPy acted as ion reservoirs that shortened ion diffusion distance. Therefore, NiNTAs@PPy electrode displayed a high specific capacitance of 474.4 F g^−1^ at 5 mV s^−1^. The PPy nanotubes also had excellent electrochemical stability with 75.3% capacitance retention after 10,000 charge–discharge cycles. The nanotube morphology and many pores and voids in PPy buffered the volumetric change of PPy and facilitated ion diffusion (Fig. 14e), which ensured excellent cycling stability.

#### Nanoplates

**Fig. 15:**
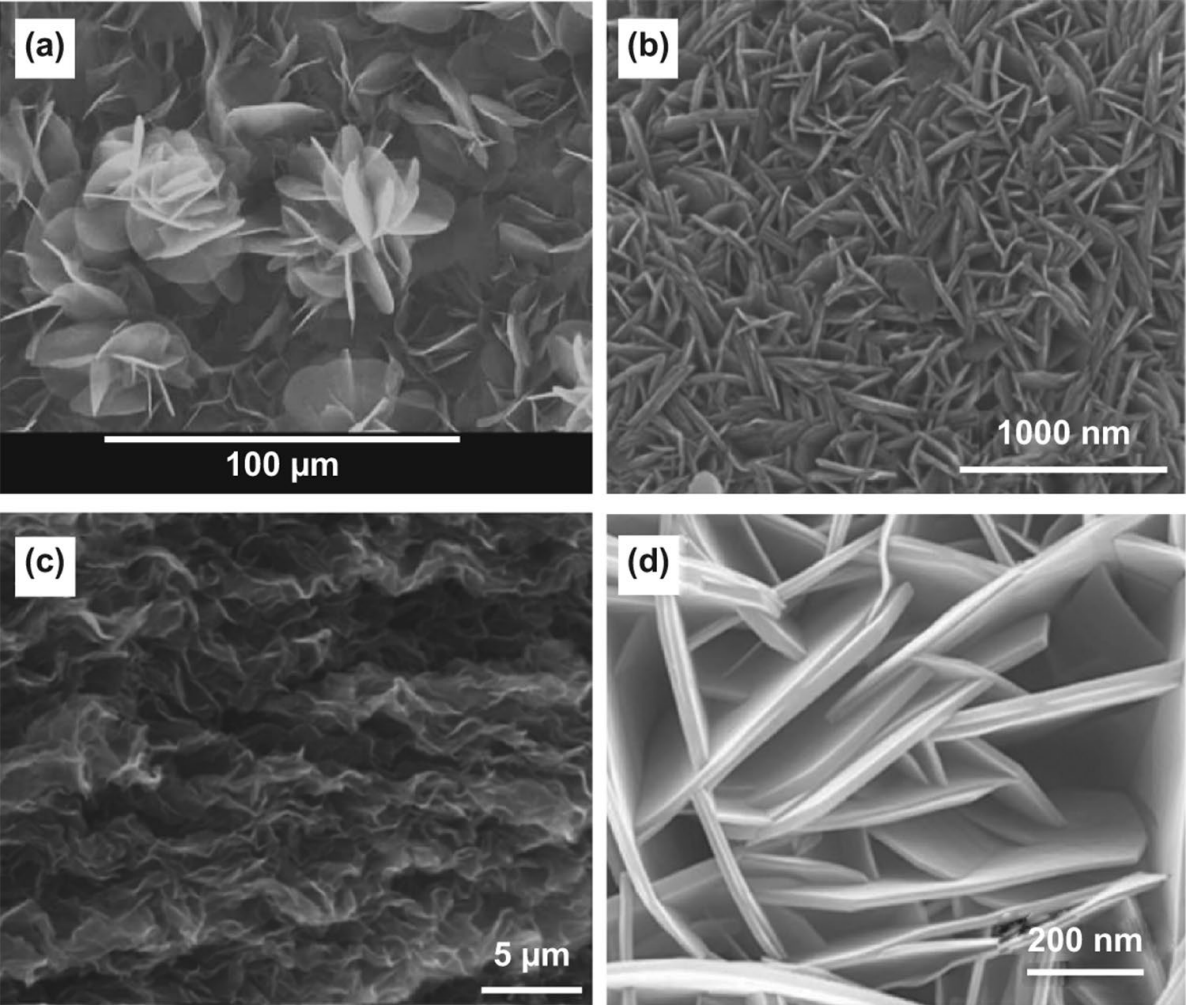
Electrodeposited two-dimensional conducting polymers. **a** Oligopyrene nanosheets; **b** PPy nanosheets; **c** PANI nanosheets on graphene sheets; **d** PEDOT thin films on CoAl layered double hydroxide nanoplates. Adapted from **a** Ref. [162], **b** Ref. [159], **c** Ref. [160], **d** Ref. [161] with permission

Electrochemically synthesized two-dimensional (2D) conducting polymers are rare because conducting polymers intrinsically prefer to grow into fibers or films. One typical example of 2D conducting polymer made by electropolymerization is pyrene nanosheets (Fig. 15a). They were grown in a mixed electrolyte containing boron trifluoride diethyl etherate (BFEE), trifluoroacetic acid (TFA), and polyethylene glycol (PEG), using a potentiostatic technique (1.2 V vs. SCE) [158]. Spectroscopy revealed that the formation of nanosheets was due to oligomer growth via α–α coupling of pyrene rings. Besides pyrene, PPy nanosheets (Fig. 15b) were synthesized using cyclic voltammetry at a high scan rate of 200 mV s^−1^ in an aqueous electrolyte containing 0.05 M pyrrole and 0.1 M KNO_3_ [159]. These PPy nanosheets interconnected with each other and assembled into a macroporous structure. It had a specific surface area of 37.1 m^2^ g^−1^ and a specific capacitance of 584 F g^−1^ at 5 mA cm^−2^.

Hard template methods can also synthesize 2D polymer materials (Fig. 15c, d). These 2D templates (e.g., graphene sheets [160] and layered double hydroxides [161]) are structural scaffolds to direct the growth of conducting polymers in 2D fashion, endowing fast ion diffusion pathways in the electrode materials that results in excellent rate capability.

#### 3D Networks

Three-dimensional (3D) conductive structures provide large ion-accessible surface areas and abundant pores compared to 2D architectures and, thus, are increasingly popular morphologies of electrochemically deposited conducting polymers. High surface area can effectively reduce the local current density and polarization in bulk electrodes and thus improve the electrodes’ charge storage kinetics. Unfortunately, ideal 3D structures composed merely of conducting polymers are challenging to acquire, due to their preferably random growth into films or fibers during electropolymerizations.

**Fig. 16:**
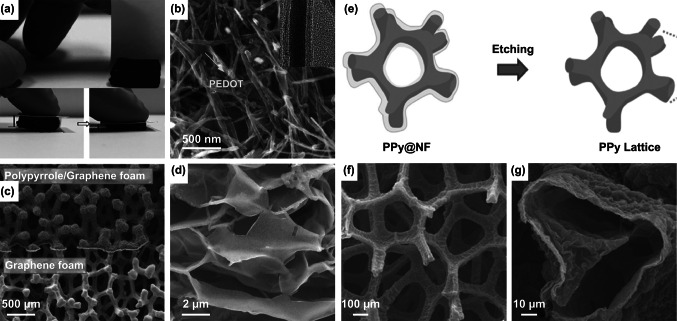
**a** Photographs and **b** SEM and TEM (inset) images of a compressible, PEDOT-coated carbon nanotube sponge. **c** SEM image of PPy-coated graphene (top) and bare graphene (bottom) foams. **d** PPy film deposited on electrochemically exfoliated graphite foil. **e** Scheme of the fabrication process of 3D PPy foam. **f**, **g** SEM images of 3D PPy foam at different magnifications. Adapted from **a**, **b** Ref. [163], **c** Ref. [164], **d** Ref. [26], **e**–**g** Ref. [165] with permission

To circumvent this challenge, researchers adopt templates to construct 3D conducting polymers. Demonstrated templates, including carbon nanotube foam [163] (Fig. 16a, b), graphene foam [164] (Fig. 18c), partial exfoliated graphite [26] (Fig. 16d), as well as Ni foam [165] (Fig. 16e–g), have been used to construct 3D polymer-based electrodes. For example, Park et al. deposited a PPy film on graphene foam (Fig. 16c) [164]. The high surface area of graphene foam and the pseudocapacitance of PPy synergistically improved the performance of the electrode. Wang et al. reported a PPy foam using a sacrificial Ni foam template (Fig. 16e) [165]. PPy was first electrodeposited on a Ni foam (Fig. 16f), and subsequently, the Ni foam was etched away, leaving a freestanding 3D PPy foam (Fig. 16g). This as-prepared 3D PPy foam was mechanically strong and highly flexible, making it a multifunctional 3D material in sensors, supercapacitors, and supports for graphene. Moreover, the freestanding 3D PPy exhibited a capacitance of 316.2 F g^−1^ at 2 mV s^−1^, and the graphene-coated 3D PPy achieved a higher capacitance of 702.9 F g^−1^ at the same scan rate. The incorporation of graphene created highly conductive surface coatings as well as increasing specific surface area from 72 to 113.4 m^2^ g^−1^.

## Metal Oxides and Hydroxides

### Manganese Oxides

Manganese oxides, particularly manganese dioxide (MnO_2_), have attracted immense interest as one of the most commercially promising pseudocapacitive materials, due to its high theoretical capacitance (~ 1000 F g^−1^), cost efficiency, source abundance, and environmental friendliness [166, 167]. A variety of chemical and electrochemical techniques have synthesized manganese oxides. Among them, anodic electrodeposition is the most time-efficient. This technique deposited MnO_*x*_ by consecutive oxidation of Mn^2+^, as illustrated in the following equations [168, 169]:

4 $${\text{Mn}}^{2 + } \to {\text{Mn}}^{3 + } + e^{ - }$$

5 $${\text{Mn}}^{3 + } + 2{\text{H}}_{2} {\text{O}} \to {\text{MnOOH}} + 3{\text{H}}^{ + }$$

6 $${\text{MnOOH}} \to {\text{MnO}}_{2} + e^{ - }$$

The nanostructures of electrodeposited MnO_*x*_ are tunable by varying the electrolyte composition, temperature, potential, and current density. For example, Feng et al. demonstrated that complexing agents such as CH_3_COO^−^ and NH_4_^+^ significantly reduced the charge transfer resistance of the electrooxidation of Mn^2+^ [166], changing the morphology of MnO_*x*_ from 2D nanosheets to 1D nanoneedles. These observations indicate that diminishing charge transfer resistance of MnO_*x*_ electrodeposition impedes its lateral growth.

**Fig. 17:**
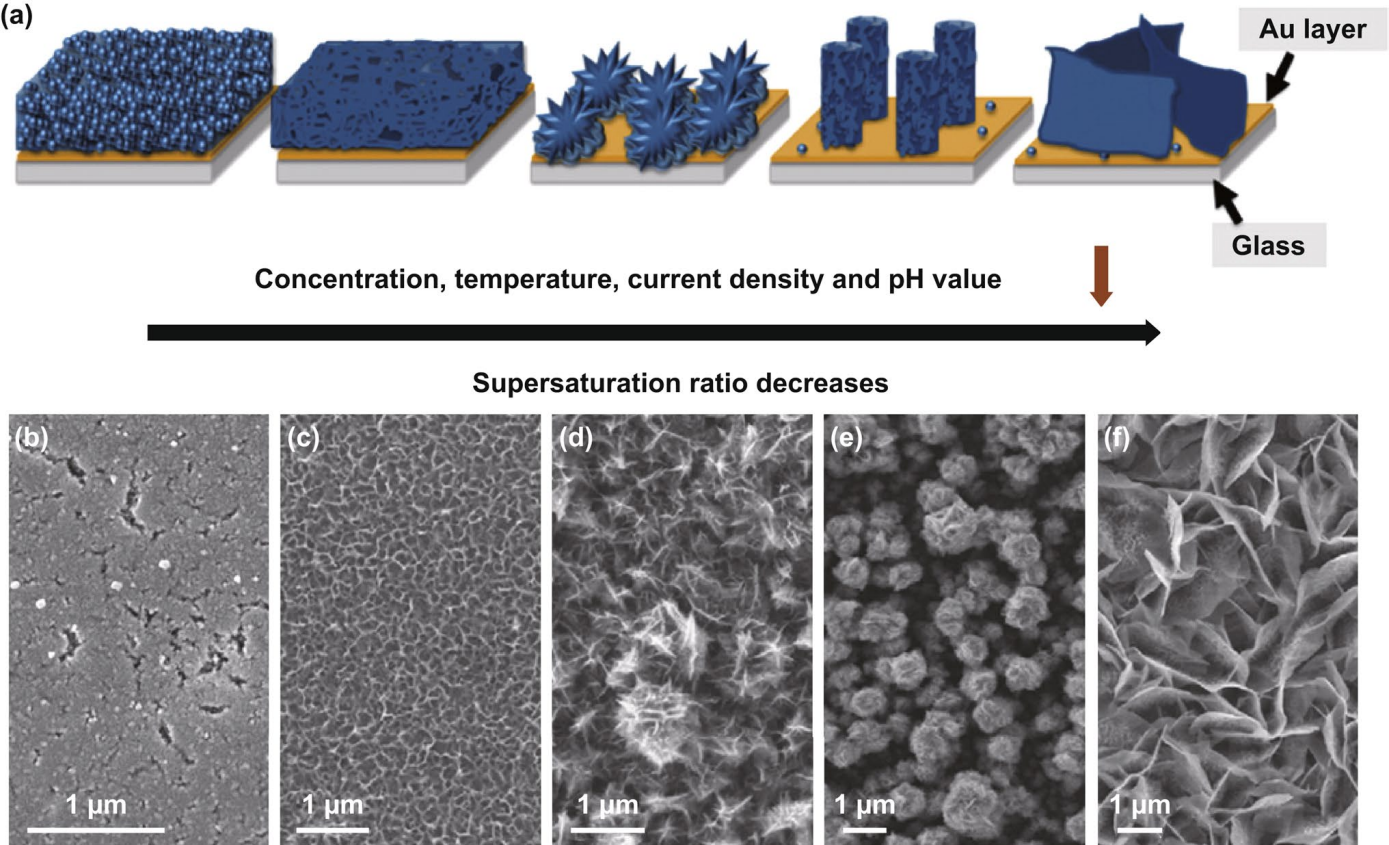
**a** Schemes of the morphological evolution of MnO_*x*_ across different electrodeposition supersaturation ratios. **b**–**d** Top-view SEM images of MnO_*x*_ electrodeposited in 0.1 M Mn(NO_3_)_2_ aqueous solutions at various current densities: **b** 20 mA cm^−2^, **c** 1 mA cm^−2^, and **d** 0.1 mA cm^−2^. **e**–**f** SEM images of MnO_*x*_ prepared in 0.0025 M Mn(NO_3_)_2_ at **e** 0.1 mA cm^−2^ and **f** 0.05 mA cm^−2^. Adapted from Ref. [170] with permission

Wei et al. proposed a theory of the supersaturation ratio of Mn^2+^ to rationalize the diverse morphologies of anodically electrodeposited MnO_*x*_ (Fig. 17a) [170]. Supersaturation ratio is defined as the ratio of the actual concentrations (or more vigorously speaking, activities) of all the ions associated with electrodeposition to the equilibrium concentrations of the same set of ions. The authors observed that high concentrations of Mn(NO_3_)_2_ and large current densities induced high supersaturation ratios that led to uniform coatings. In contrast, low concentrations of Mn(NO_3_)_2_ and small current densities favored epitaxial growth into interconnected nanosheets (Fig. 17b–f). These different morphologies were associated with the number of nucleates formed at the beginning of electrodeposition. High supersaturation ratios yielded abundant nucleation sites that suppressed epitaxial growth. Parameters that lowered the supersaturation ratio decreased the number of nucleation sites and favored the formation of nanostructures.

#### Nanorods and Nanotubes

**Fig. 18:**
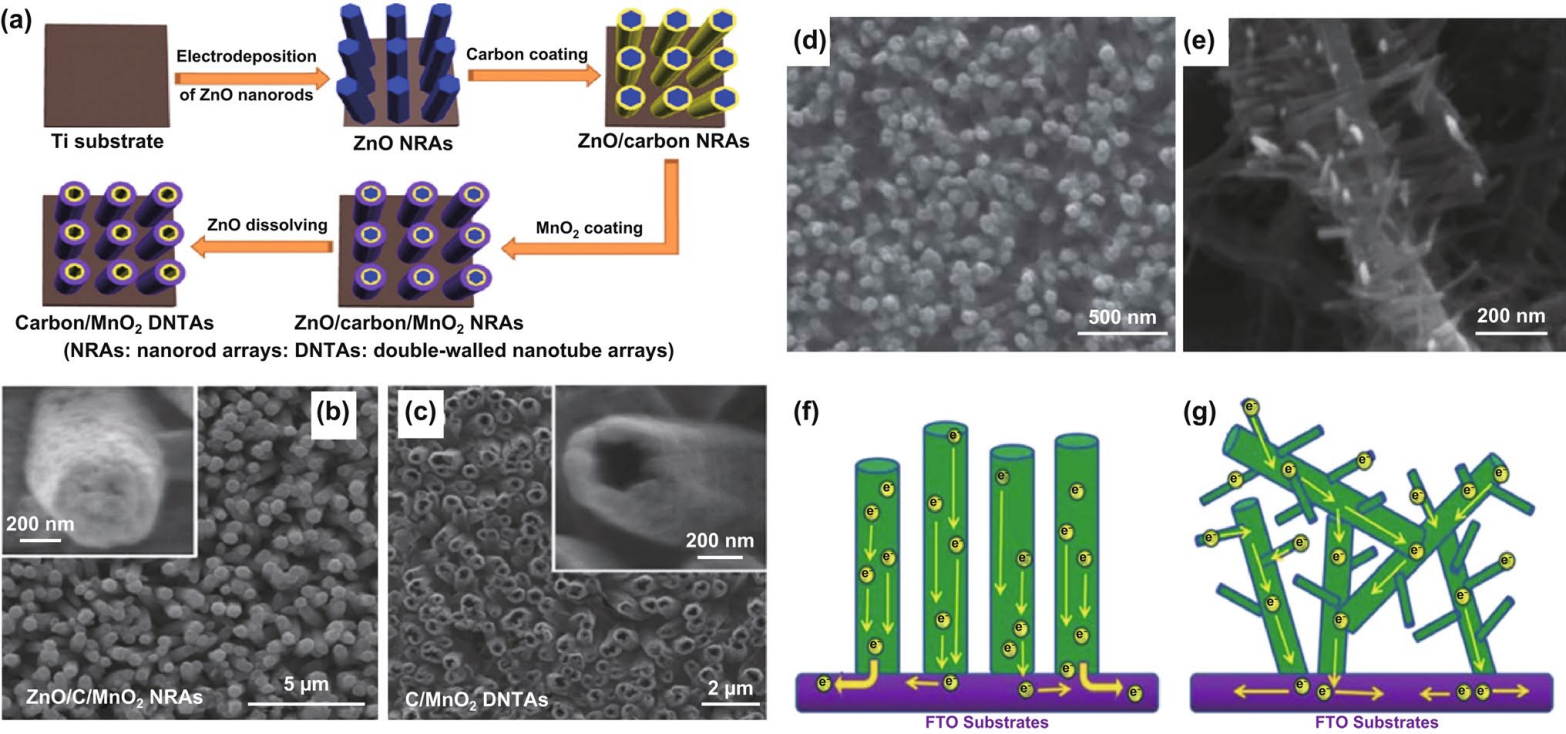
**a** Schemes of the synthesis procedures of double-walled MnO_2_ nanotube arrays on carbon cloth. **b** SEM image of ZnO/C/MnO_2_ nanorod arrays. Inset: Magnified view of a single nanorod. **c** SEM image of C-coated MnO_2_ double-walled nanotubes. Inset: Magnified view showing a C-coated MnO_2_ nanotube. **d**, **e** SEM images of MnO_*x*_ **d** nanorods and **e** herringbones. **f**, **g** Schemes of the charge transfer pathways in **f** MnO_*x*_ nanorod arrays and **g** MnO_*x*_ herringbones. Adapted from **a**–**c** Ref. [173] and **d**–**g** Ref. [180] with permission

Templating is a typical strategy to prepare 1D MnO_*x*_ nanostructures [171, 172]. ZnO nanorods [173], anodized alumina [174, 175], hydrogenated TiO_2_ nanorods [176], and silicon square pillars [177] are reported templates for electrodepositing MnO_*x*_ nanorod or nanotube arrays. For example, Li and coworkers synthesized double-walled carbon/MnO_2_ nanotube arrays using ZnO nanorods as sacrificial templates [173] (Fig. 18a). First, ZnO nanorod arrays were grown on Ti plates via electrodeposition, and thin layers of carbon were coated on the nanorods to render the ZnO nanorods electrically conductive. Afterward, a uniform MnO_2_ film was electrodeposited on the carbon-coated ZnO nanorod arrays (Fig. 18b). Finally, dissolving the ZnO nanorod arrays using 0.5 M NaOH solution generated the double-walled carbon/MnO_2_ nanotube arrays (Fig. 18c). These double-walled nanotubes displayed a high specific capacitance of 793 F g^−1^ at 1.5 A g^−1^, and a rate capability of 83% when the scan rate increased from 5 to 50 mV s^−1^. The excellent electrochemical performances were ascribed to factors including: (1) the hollow structure of the nanotube arrays exposed plentiful active sites of MnO_2_ and provided ions with fast diffusion pathways; (2) the conformal carbon coating served as electron transport expressways, minimizing capacitance loss at elevated scan rates; and (3) the high weight fraction of MnO_2_ (~ 98.94 wt%) in the electrodes was beneficial to achieve high specific capacitance and energy density.

Besides templating, template-free methods could also synthesize 1D nanostructured MnO_*x*_. These methods are time-efficient and can synthesize products of high purity because they lift the needs for template incorporation and removal [178, 179]. For example, Lu and coworkers demonstrated that adding dimethyl sulfoxide (DMSO) in the deposition solution of MnO_2_ led to MnO_2_ nanorod arrays without any templates [180]. They applied a constant anodic current of 0.2 mA cm^−2^ at 70 °C and used aqueous electrolytes containing 0.01 M manganese(II) acetate, 0.02 M ammonium acetate, and 10 wt% DMSO. The resultant MnO_2_ nanorods had diameters between 70 and 100 nm, and lengths up to ~ 1.5 μm (Fig. 18d). Electrodeposition without DMSO only yielded MnO_2_ herringbones (Fig. 18e). Though the authors did not justify how DMSO changed the deposit morphology, we hypothesized that the addition of DMSO reduced the supersaturation ratio of Mn^2+^, and thus promoted epitaxial growth of MnO_2_ into nanorods. The specific capacitance of the MnO_2_ nanorod array was 660.7 F g^−1^ at 10 mV s^−1^, which was ~ 100 F g^−1^ higher than that of the herringbone structured MnO_2_ (564.3 F g^−1^). This capacitance discrepancy was associated with the morphology: The ordered vertically aligned nanorods, compared with the herringbones, reduced the tortuosity and distances for electron transport, which boosted capacitance (Fig. 18f, g).

#### Nanosheets and Nanoplates

**Fig. 19:**
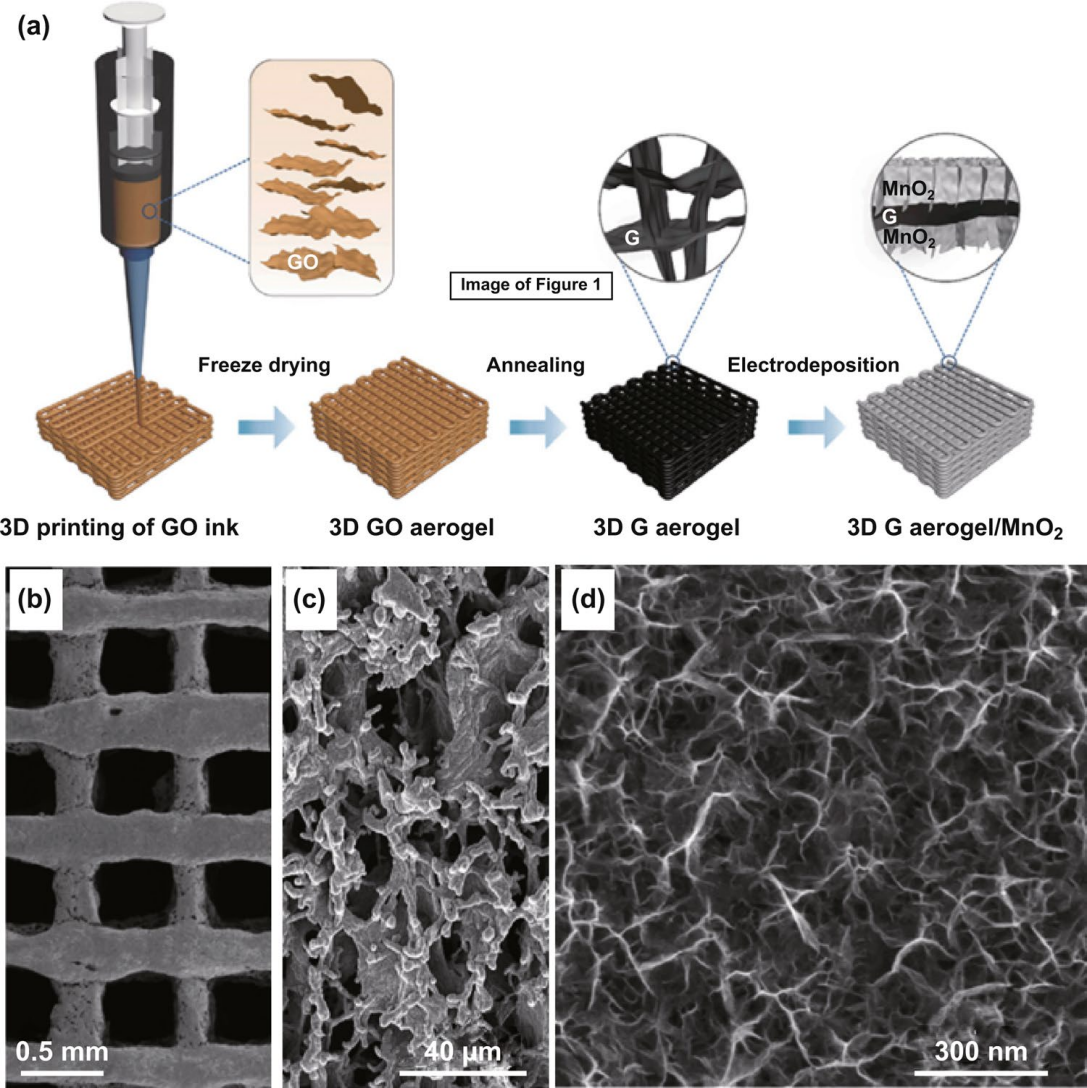
**a** Schemes of the synthesis procedures of MnO_2_ nanosheets deposited on 3D printed graphene aerogel lattices. **b** A top-view SEM image of MnO_2_-coated 3D printed graphene aerogel. **c**, **d** SEM images of the deposited MnO_2_ nanosheets at two magnifications. Adapted from Ref. [196] with permission

Ultrathin 2D MnO_*x*_ nanosheets were other common morphologies of electrodeposited MnO_*x*_ [181–185]. Anodic deposition is widely demonstrated to prepare MnO_*x*_ nanosheets [186–195]. For example, Yao et al. deposited MnO_2_ nanosheets onto 3D printed graphene aerogel lattices through an anodic galvanostatic deposition at 10 mA cm^−2^ (Fig. 19a) [196]. The outstanding property of these 3D printed MnO_2_/graphene composite electrodes was their uncompromised electrochemical performance at MnO_2_ mass loadings as high as 182.2 mg cm^−2^. The areal capacitance scaled linearly with the thickness of the electrode, reaching 44.13 F cm^−2^ at 0.5 mA cm^−2^ in 3 M LiCl aqueous electrolytes at a thickness of 4.0 mm (MnO_2_ mass loading 182.2 mg cm^−2^). This linear relationship indicated that the charge storage process of the electrode was not under diffusion control or limited by ion percolation even at ultrahigh mass loadings and thicknesses. This merit was attributed to the 3D-printed graphene lattices with macropores of 5–50 μm pores (Fig. 19b, c), which promoted the uniform deposition of MnO_2_ and opened up wide ion diffusion pathways throughout the entire electrodes.

In addition to anodic electrodeposition, cathodic electrodeposition also synthesizes 2D MnO_*x*_ nanosheets. For example, Beyazay et al. used a chronoamperometry technique to deposit Mn_3_O_4_ hexagonal nanosheets on graphene paper. This electrode delivered a maximal specific capacitance of 546 F g^−1^ at 0.5 A g^−1^. Interestingly, the capacitance increased about 1.5 times after being charged and discharged for 10,000 times. XPS analysis after the stability test found that the average valence of Mn raised from 2.7 to 3.2, indicating that part of Mn_3_O_4_ was oxidized to MnO_2_. Besides the valence change, some hexagonal nanosheets transformed into particles and needles. These results suggested that the hexagonal Mn_3_O_4_ nanosheets were both chemically and structurally unstable during long-term cycling tests.

#### Hierarchical Structures

**Fig. 20:**
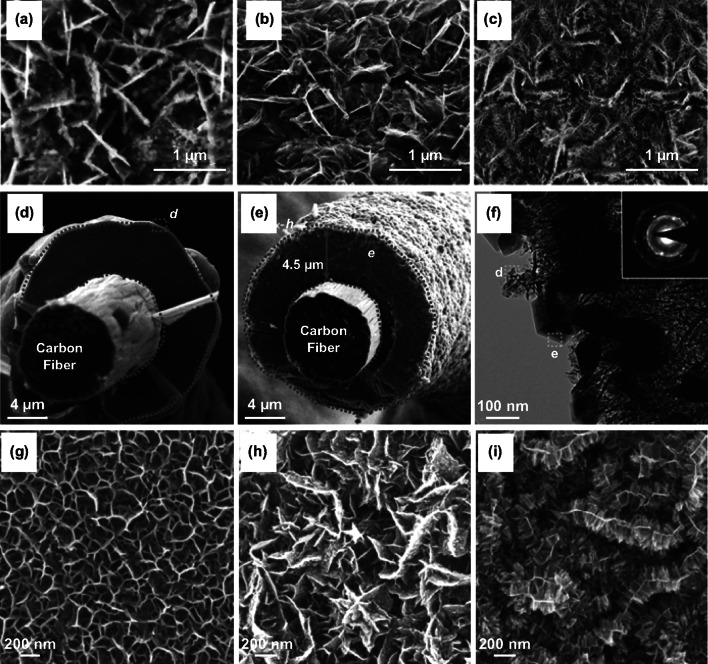
**a**–**c** SEM image of **a** Mn_3_O_4_ nanowall arrays, **b** intermediates during electrochemical oxidation, and **c** hierarchical Na_0.5_MnO_2_ nanowall arrays. **d**, **e** SEM images of **d** as-deposited and **e** hydrothermally treated MnO_*x*_ thick layer on carbon fiber. **f** TEM image of hydrothermally treated MnO_*x*_. **g**–**i** SEM images of MnO_2_ deposited at **g** 25 °C, **h** 40 °C, and **i** 60 °C. Adapted from **a**–**c** Ref. [14]; **d**–**f** Ref. [197]; **g**–**i** Ref. [50] with permission

Hierarchical MnO_*x*_ integrates nanostructures of different dimensions, e.g., 1D nanorod, 2D nanosheet, and 2D nanoplate. Electrodepositing hierarchical structures often begins with one specific structure. For example, Jabeen et al. synthesized Mn_3_O_4_ nanosheet-on-nanowall arrays via a cathodic potentiostatic method (− 1.8 V vs. Ag/AgCl) in an aqueous solution containing 0.1 M manganese acetate and 0.1 M sodium sulfate. High-resolution scanning electron microscopy revealed that these nanowalls were composed of interconnected nanoparticles (Fig. 20a) [14]. After 500 cycles of electrochemical oxidation in 10 M sodium sulfate aqueous solutions, the nanoparticles disappeared, and nanosheets appeared on the surface of the nanowalls, assembling the nanosheet-on-nanowall hierarchical structure (Fig. 20b, c). Meanwhile, the composition of the electrode changed from Mn_3_O_4_ to Na_0.5_MnO_2_. The hierarchically structured Na_0.5_MnO_2_ exhibited a specific capacitance of 366 F g^−1^ at 1 A g^−1^. Besides, the redox peak of Na_0.5_MnO_2_ at ~ 0.96 V vs. Ag/AgCl extended the upper limit potential to approximately 1.3 V vs. Ag/AgCl, enabling the development of aqueous-based supercapacitors with high voltages and energy densities.

The most significant characteristic of hierarchical structures is their capability to maintain excellent electrochemical performance at MnO_*x*_ mass loadings exceeding 10 mg cm^−2^. Increasing the mass loadings of MnO_*x*_ (and other pseudocapacitive materials) has become a trend in recent years, due to the consideration of practicality. Unfortunately, the capacitances of poorly conductive pseudocapacitive materials, including MnO_*x*_, are greatly compromised when enhancing their mass loadings, particularly under fast charge and discharge rates. Hierarchical structures could resolve this challenge. For example, Song et al. developed an Ostwald ripening strategy that improved the rate capability of electrodeposited MnO_*x*_ thick films with a high mass loading of ~ 10 mg cm^−2^ (Fig. 20d–f) [197]. The authors first coated carbon fibers with MnO_*x*_ films of ~ 4.5 µm thick using a constant current of 10 mA cm^−2^ in 0.1 M manganese acetate aqueous solutions (Fig. 20d). They then hydrothermally treated the electrodeposited MnO_*x*_ at 90 °C, which appreciably altered the morphology of the MnO_*x*_ films. First, many crystalline MnO_*x*_ nanosheets formed on the surface. Second, the porosity of the MnO_*x*_ core increased (Fig. 20e, f). The porous MnO_*x*_ core and oxide shell together constituted a core–shell hierarchical structure. The crystalline surface ensured good electrical conductivity, and the porous MnO_*x*_ core sped up ion diffusion. Therefore, the electrode exhibited improved rate performance even at a high mass loading of 10 mg cm^−2^. Recently, Huang et al. have demonstrated a facile electrochemical technology that synthesized a nanorod-on-nanosheet hierarchical structure. The structure consisted of primary two-dimensional *ε*-MnO_2_ nanosheets and secondary one-dimensional *α*-MnO_2_ nanorod arrays (Fig. 20g–i) [50]. Morphology studies indicated that elevating the deposition temperature to 60 °C and 80 °C added nucleation sites on the as-formed nanorods, which favored the secondary growth of nanorods. This hierarchical electrode had a high MnO_2_ mass loading of 10 mg cm^−2^ and delivered a high areal capacitance of 3.04 F cm^−2^ at 3 mA cm^−2^. Significantly, the areal capacitance maintained at 1.9 F cm^−2^ at 30 mA cm^−2^. The authors ascribed this excellent rate capability performance to two factors: First, the multiple connections between the nanorods and nanosheets created fast avenues for electron transport. Second, the voids among the nanorods and nanosheets throughout the hierarchical structure facilitated electrolyte ion percolation and ion diffusion.

### Vanadium Oxides

Vanadium oxides (VO_*x*_), mainly vanadium pentoxide (V_2_O_5_), have the advantages of high specific capacitance (multiple electron reaction, e.g., from + 3 to + 5), low cost, ease of fabrication, as well as wide potential windows [37, 198–202]. Electrochemical technologies are particularly suitable for synthesizing VO_*x*_ of diverse morphologies, crystal structures, and valence states [203–209]. In aqueous electrolytes, vanadium oxide is typically synthesized from the oxidation of vanadium-containing ions with the aid of water molecules. For example, oxovanadium(IV) cations, VO^2+^, are electrooxidized to high-valence vanadium oxides (e.g., V_2_O_5_) through the following equation [208]:

7 $$2{\text{VO}}^{2 + } + 3{\text{H}}_{2} {\text{O}} \to {\text{V}}_{2} {\text{O}}_{5} + 6{\text{H}}^{ + } + 2e^{ - }$$

For example, Xie et al. demonstrated that the pH value and composition of acetate salts (CH_3_COONa, CH_3_COOLi, CH_3_COOK) were critical in tuning the deposition rate, crystal structure, and morphology of VO_*x*_ [209]. Drosos et al. studied the effects of the deposition current density on the morphology and electrochemical performance of V_2_O_5_ coatings on indium-doped tin oxide glass substrates in 1 M LiClO_4_ polypropylene carbonate electrolytes. The V_2_O_5_ film deposited at 1 mA cm^−2^ exhibited the highest capacitance owing to its roughest surface [210].

**Fig. 21:**
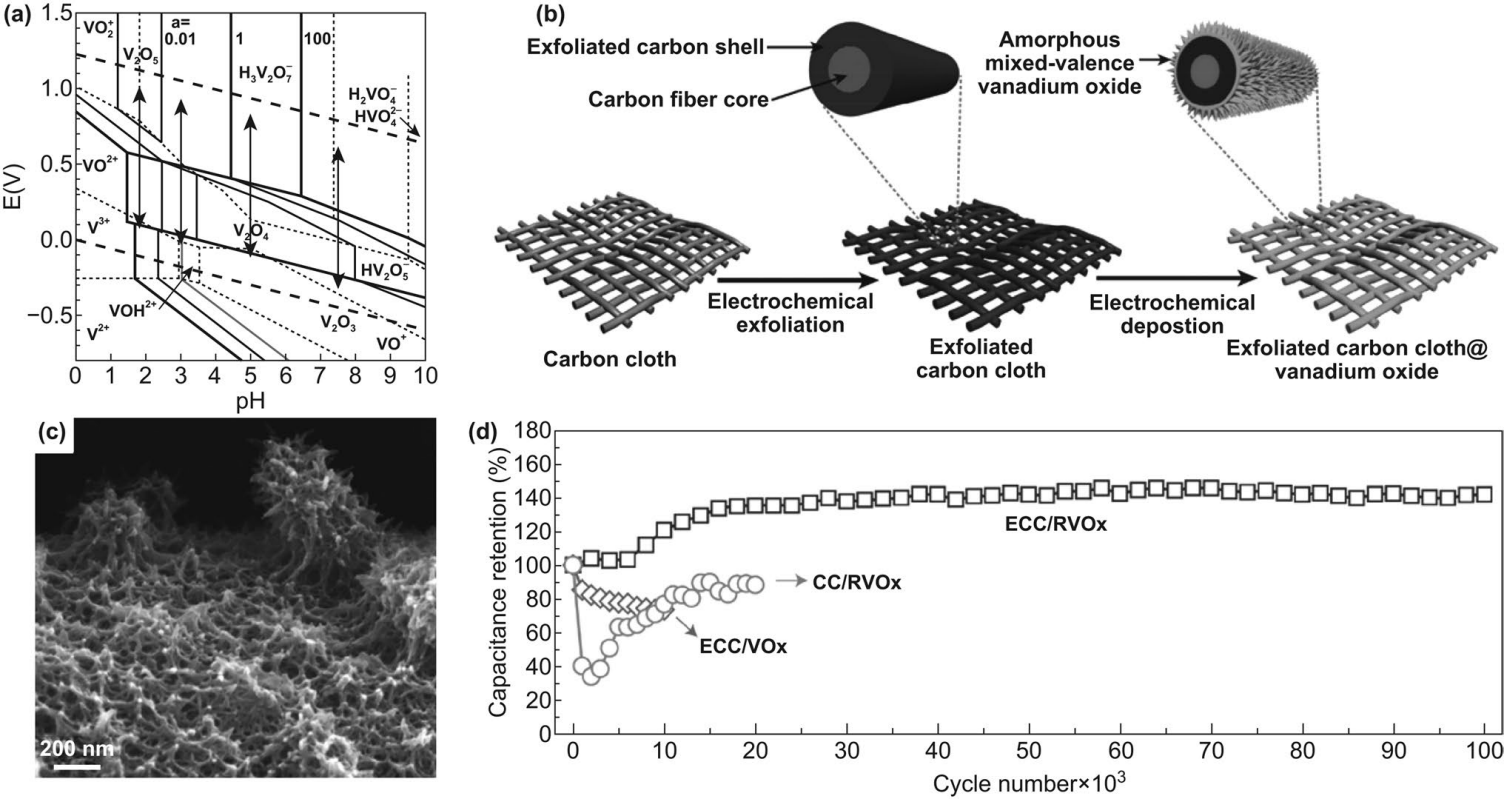
**a** *E*–pH diagram of vanadium oxide–water system with various V-based species at activities (the letter “a” in the figure) of 0.01, 1 and 100. **b** Schemes of the synthesis steps, **c** SEM image, and **d** cycling stability of amorphous, mixed-valence vanadium oxide (RVO_*x*_) deposited on exfoliated carbon cloth fibers. Adapted from **a** Ref. [199] and **b**–**d** Ref. [37] with permission

Electrodeposited VO_*x*_ with mixed V valences are platforms for studying the interplays between V valence and cycling stability. Recent studies indicated that the performance degradation was linked to dissolution, structural pulverization, and irreversible phase transition of VO_*x*_ [34, 37, 199]. Investigating the cycling behavior of electrodeposited VO_*x*_ electrodes in various aqueous environments, Engstrom and Doyle concluded that the formation of water-soluble V-containing species, including H_2_VO_4_^−^, HVO_4_^2−^, HV_2_O_5_^−^, VO^2+^, HVO^2+^, and VO^+^ (Fig. 21a), was the primary cause of capacitive decay of VO_*x*_ during extensive cycling tests [199]. Though chemical strategies such as surface coating [211, 212] and electrolyte pH value tuning [199] minimized dissolution of VO_*x*_, the altered electrode kinetics usually compromised capacitance. To circumvent these limitations, Song et al. utilized a potentiostatic electrochemical method to tune the V valence in VO_*x*_ and achieved record-high cycling stability without capacitive decay over 100,000 cycles (Fig. 21b–d) [37]. VO_*x*_ nanorods were first electrodeposited on electrochemically exfoliated carbon cloth fibers using cyclic voltammetry between − 1.5 and 1.4 V vs. SCE (Fig. 21c). The authors then reduced the as-deposited VO_*x*_ at a constant potential of − 1.5 V vs. SCE for 1 min. This reduction raised the V^4+^/V^5+^ ratio from 0.4 to an optimal value of around 0.5. The optimized V^4+^/V^5+^ ratio in VO_*x*_ effectively suppressed the chemical dissolution of VO_*x*_. Meanwhile, the firm anchoring of the reduced VO_*x*_ nanorods on oxygenated, exfoliated carbon cloth fibers via C–O–V bonds retained the structural integrity of VO_*x*_. Both factors contributed to the excellent cycling stability (ECC/RVO_*x*_ in Fig. 21d).

**Fig. 22:**
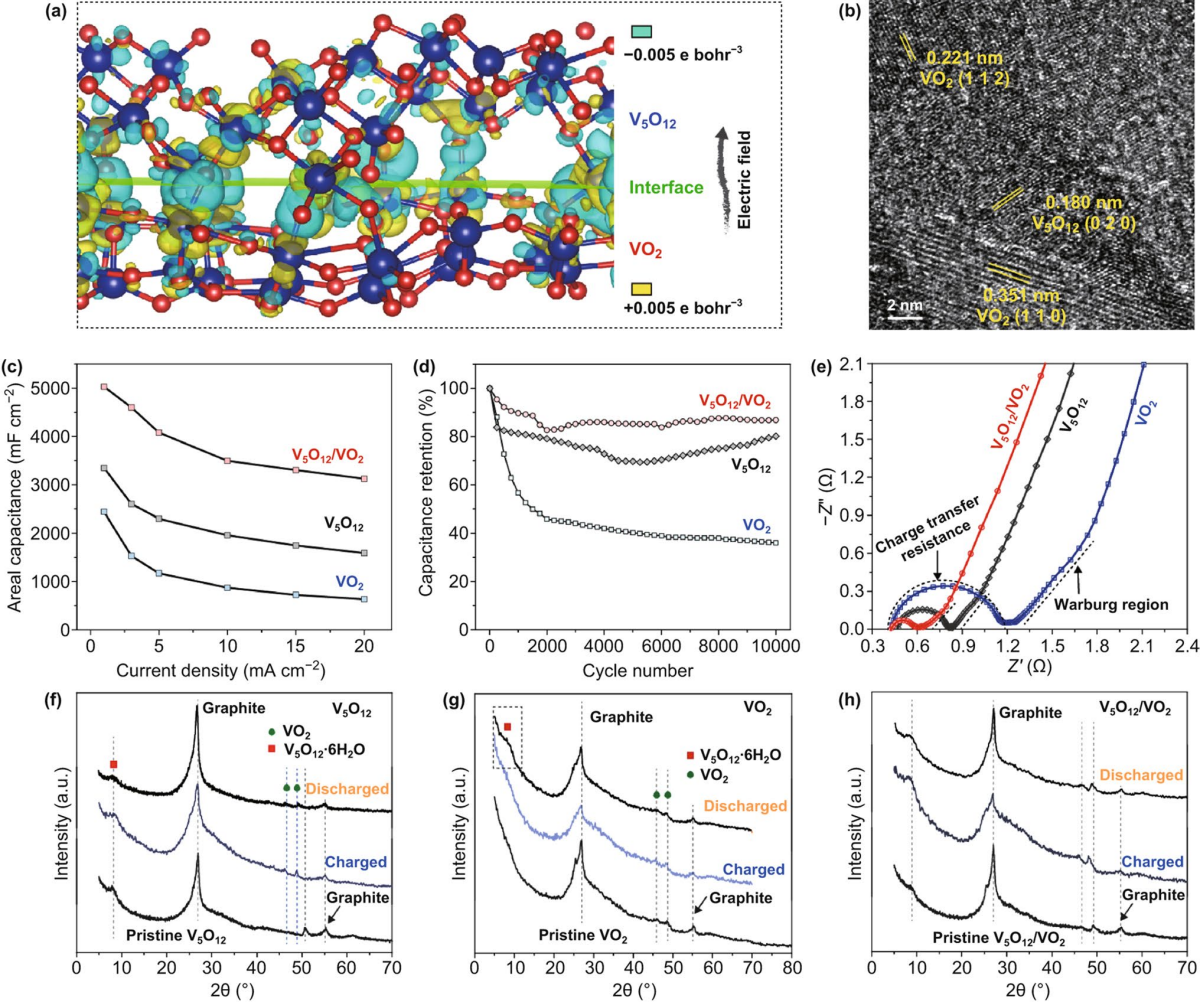
**a** Charge density distribution along the interface of V_5_O_12_/VO_2_. **b** TEM image of V_5_O_12_/VO_2_ junction. **c** Areal capacitance of V_5_O_12_/VO_2_, V_5_O_12_, and VO_2_ as a function of current density. **d** Cycling stability of V_5_O_12_/VO_2_, V_5_O_12_, and VO_2_ electrodes in 3 M LiCl aqueous electrolyte. **e** Nyquist plots of V_5_O_12_/VO_2_, V_5_O_12_, and VO_2_. **f**–**h** Ex situ XRD patterns of **f** V_5_O_12_, **e** VO_2_ and **h** V_5_O_12_/VO_2_ without charging (pristine), charged (− 1 V), and discharged (0 V). Reproduced from Ref. [34] with permission

The easy valence tuning of VO_*x*_ by electrochemical techniques allowed the synthesis of heterojunctions between VO_*x*_ of two valences. These configurations help facilitate electron transfer within VO_*x*_ electrodes having large thicknesses or high mass loadings [34]. For instance, Dong et al. performed a density functional theory (DFT) calculation and discovered that a built-in electric field formed at the V_5_O_12_/VO_2_ heterojunction. The charge redistribution between the two oxides led to an electric field pointing from VO_2_ to V_5_O_12_ (Fig. 22a) [34]. This built-in electrical filed facilitated electron transfer and modulated ion absorption during charge storage processes, which improved electrochemical performance. Inspired by this calculation result, the authors adopted cyclic voltammetry (− 1.5 to 1.5 V vs. SCE) to electrodeposit V_5_O_12_/VO_2_ nanorods on an exfoliated graphite substrate. V_5_O_12_ first formed during the positive scan, and it was partially reduced to VO_2_ in the subsequent negative scan (Fig. 22b). V_5_O_12_/VO_2_ with a high mass loading of about 10.8 mg cm^−2^ delivered a high areal capacitance of 5.03 F cm^−2^ (465 F g^−1^) at 1 mA cm^−2^, outperforming pure V_5_O_12_ and VO_2_ (Fig. 22c). Significantly, V_5_O_12_/VO_2_ also exhibited enhanced cycling stability compared to V_5_O_12_ and VO_2_ alone (Fig. 22d). Two reasons could account for this stability enhancement. First, EIS indicated that V_5_O_12_/VO_2_ exhibited reduced charge transfer resistance due to the heterojunction (Fig. 22e). Second, ex situ XRD confirmed that V_5_O_12_/VO_2_ underwent no phase transitions after charging and discharging, while the phases of V_5_O_12_ and VO_2_ changed dramatically (Fig. 22f–h). The reduced resistance and suppressed phase change of V_5_O_12_/VO_2_ both enhanced cycling stability.

### Molybdenum Oxides

Molybdenum oxides (MoO_*x*_) are another group of electrodeposited pseudocapacitive materials [213–215]. Unlike MnO_*x*_ discussed in the previous sections, MoO_*x*_ is acid-resistant and thus can work in acidic electrolytes [216]. Besides surface redox reactions, some MoO_*x*_, e.g., α-MoO_3_, possess layered structures that allow ion insertion and de-insertion that contribute intercalative pseudocapacitance [217]. Specifically, α-MoO_3_ can accommodate up to 1.5 Li^+^ per Mo, having a high theoretical capacity of 1117 mAh g^−1^ [218].

MoO_*x*_ is usually deposited by cathodic electrodeposition in aqueous electrolytes containing molybdates (e.g., sodium molybdate and ammonium molybdate) [38], iso-/peroxo-polymolybdates [213], and ammonium paramolybdates [219]. Electrodeposited MoO_*x*_ are typically quasi-amorphous, non-stoichiometric oxide films [38, 220–222]. Its composition, structure, and electrochemical performance of the deposited MoO_*x*_ materials highly depend on electrolyte composition, pH value, and magnitudes of the applied current density and voltage [223–225].

**Fig. 23:**
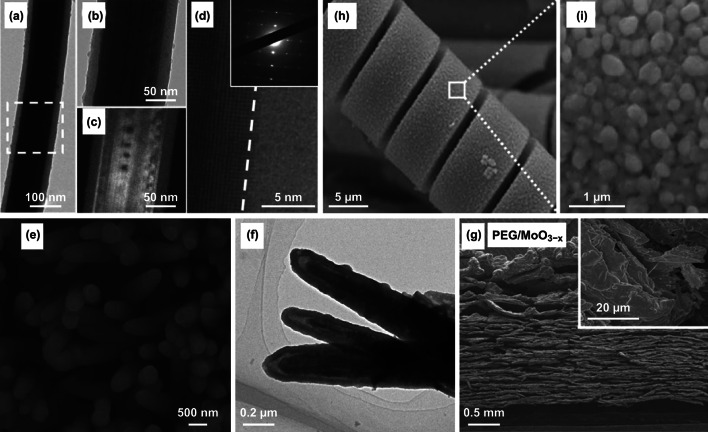
**a**–**c** TEM images of a MoO_3−*x*_-coated WO_3−*x*_ nanowire: **a**, **b** bright and **c** dark fields. **d** High-resolution TEM image of a MoO_3−*x*_-coated WO_3−*x*_ nanowire. Inset: Selected electron diffraction pattern of WO_3−*x*_. **e** An SEM and **f** TEM images of ZnO@MoO_3_ core–shell structure. **g** A SEM image of MoO_3−*x*_ film deposited on an exfoliated graphite substrate. **h**, **i** SEM images of the helical porous MoO_2_ with different magnifications. Adapted from **a**–**d** Ref. [226], **e**, **f** Ref. [227], **g** Ref. [228], **h** Ref. [229] with permission

Nanostructured substrates with large ion-accessible surface areas are preferred electrodeposition scaffolds for MoO_*x*_. For example, a ~ 18-nm-thick layer of MoO_*x*_ (3 mg cm^−2^) was deposited on tungsten oxide nanowires, forming a WO_3–*x*_/MoO_3–*x*_ core/shell structure (Fig. 23a–d) [226]. This core/shell electrode delivered an areal capacitance of 500 mF cm^−2^. Li et al. electrodeposited a 40-nm-thick MoO_3_ layer (2.43 mg cm^−2^) on ZnO nanorod arrays (Fig. 23e, f). The ZnO-supported MoO_3_ displayed a specific capacitance of 241 F g^−1^ at 5 mV s^−1^ and 198 F g^−1^ at 100 mV s^−1^ [227]. Liu et al. demonstrated that functionalized, partially exfoliated graphite foil substrates could support MoO_*x*_ films with high mass loadings (18.4 mg cm^−2^) (Fig. 23g) [228]. The exfoliated graphene sheets and the laminar structure of the graphite base addressed the negative impact of the poor electrical conductivity of the atop MoO_*x*_. This highly conductive carbon-based structure permitted efficient ion diffusion and fast electron transport. Besides, the O-functional groups on the exfoliated graphite foil formed covalent C–O–Mo bonds with MoO_3_, which served as bridges that permitted fast charge transport from MoO_*x*_ to the substrate. All the above factors led to excellent rate capability: The optimized electrode with a high MoO_*x*_ mass loading of 15.4 mg cm^−2^ delivered an areal capacitance of 4.34 F cm^−2^ at 1 mA cm^−2^ and retained 67.8% of the initial capacitance at 20 mA cm^−2^.

Films deposited onto fibers can develop unique morphologies, such as helical cracks demonstrated by Lu et al. They utilized a combined electrochemistry–annealing strategy and deposited hierarchical porous MoO_2_ films composed of mesoporous nanoparticles on carbon cloth fibers (Fig. 23h, i) [229]. First, mixed-valence MoO_*x*_ was electrochemically deposited on carbon cloth by reducing Mo_7_O_24_^6−^:

8 $$6{\text{NH}}_{4}^{ + } + {\text{Mo}}_{7} {\text{O}}_{24}^{6 + } + \left( {18 - 7x} \right){\text{H}}_{2} {\text{O}} + \left( {42 - 14x} \right)e^{ - } \to 7{\text{MoO}}_{x} + 6{\text{NH}}_{3} + \left( {42 - 14x} \right){\text{OH}}^{ - }$$

Subsequently, the as-formed MoO_*x*_ was annealed in NH_3_ at 700 °C to obtain MoO_2_ films. Scanning electron microscopy revealed that the MoO_2_ films exhibited helical cracks of ~ 100–200 nm wide. Owing to the helical openings that reduced the dead volume of MoO_2_, the electrode delivered a high areal capacitance of 175 mF cm^−2^ at 1.43 mA cm^−2^ in Na_2_SO_4_ aqueous electrolytes. The formation mechanism of the helical cracks remained unclear, but we hypothesized that it might be associated with dehydration of the electrodeposited films during the thermal treatment in NH_3_. The cracks were initiated by volume shrinkage during annealing and propagated around carbon fibers following a helical path.

Like other pseudocapacitive materials, electrodeposited MoO_*x*_ exhibits high capacitance, but unsatisfactory cycling instability. To extend the life span of electrodeposited MoO_*x*_, Cai et al. reported a potential window tuning strategy for MoO_*x*_ [38]. They discovered that the potential window within − 1 to − 0.4 V vs. SCE permitted the redox reaction associated with Mo^4+^ and Mo^5+^ and prevented the formation of Mo^6+^. This feature resulted in no capacitance decay in 30,000 cycles. In contrast, the same electrode scanned between − 1.0 and 0 V vs. SCE irreversibly generated Mo^6+^ and its capacitance decayed by more than 25% within 500 charge–discharge cycles. Electrochemical impedance spectroscopy revealed that the accumulation of Mo^6+^ increased the combined series resistance of MoO_*x*_, which made the electrode electrically insulating. Besides, optimizing the composition of electrodeposited MoO_*x*_ materials via electrochemical technologies could provide new opportunities for enhancing their durability, as already proved to be successful in stabilizing VO_*x*_ [34].

### Tungsten Oxides

Another transition metal oxide is tungsten oxides (WO_*x*_) [230–233]. WO_*x*_ is usually deposited by cathodic deposition (e.g., applying a CV scan of − 0.5–0 V vs. SCE [234]) in aqueous electrolytes containing peroxy-tungstate species (e.g., ${\text{W}}_{2} {\text{O}}_{11}^{2 - }$) [235–237]:

9 $${\text{W}}_{2} {\text{O}}_{11}^{2 - } + \left( {2 + x} \right){\text{H}}^{ + } + xe^{ - } \to 2{\text{WO}}_{3} + \frac{2 + x}{2}{\text{H}}_{2} {\text{O}} + \frac{8 - x}{4}{\text{O}}_{2}$$

10 $${\text{W}}_{2} {\text{O}}_{11}^{2 - } + 2.36{\text{H}}^{ + } + 0.94{\text{H}}_{2} {\text{O}} + 0.36e^{ - } \to 2{\text{H}}_{0.12} {\text{WO}}_{3} \cdot {\text{H}}_{2} {\text{O}} + 1.97{\text{O}}_{2}$$

**Fig. 24:**
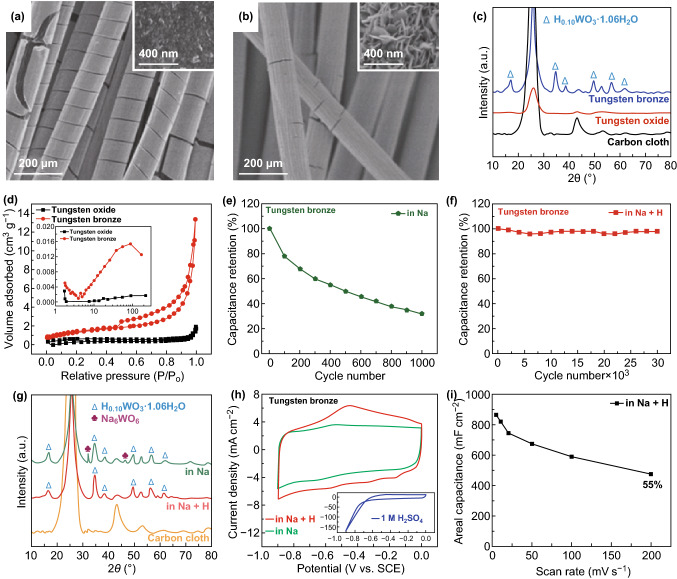
**a**, **b** SEM images of **a** electrodeposited tungsten oxide and **b** tungsten bronze films on carbon cloth. **c** XRD patterns of tungsten oxide, tungsten bronze, and carbon cloth. **d** N_2_ adsorption–desorption isotherms of tungsten oxide and tungsten bronze. Inset: pore size distributions. **e**, **f** Cycling stability of tungsten bronze in **e** 1 M Na_2_SO_4_ and **f** Na_2_SO_4_/H_2_SO_4_ (1 M/0.01 M) mixed aqueous electrolytes. **g** XRD patterns of tungsten bronze after cycled in Na_2_SO_4_ and Na_2_SO_4_/H_2_SO_4_. **h** CV curves of tungsten bronze in Na_2_SO_4_ (green), Na_2_SO_4_/H_2_SO_4_ (red), and H_2_SO_4_ (blue) aqueous electrolytes. **i** Rate capability of tungsten bronze in Na_2_SO_4_/H_2_SO_4_. Adapted from Ref. [234] with permission

Most as-deposited WO_*x*_ materials are amorphous with stacked, hydrated nanoparticles, WO_*x*_·*n*H_2_O [234, 238, 239]. Thermal annealing of WO_*x*_·*n*H_2_O at temperatures above 400 °C in air dehydrates the nanoparticles to crystalline WO_*x*_ [239, 240]. However, the annealing treatment usually triggers particle coalescence that decreases surface area. To circumvent this problem, Sun et al. developed an electrochemical post-crystallization process to convert electrodeposited amorphous, mixed-valence WO_*x*_ into crystalline tungsten bronze H_*x*_WO_3_ (Fig. 24a–c) [234]. The electrochemical crystallization process turned the non-porous film into highly porous nanosheets, enhancing specific surface area (Fig. 24d).

Changing electrolyte composition to extend the lower potential limit of WO_*x*_ electrodes is necessary to increase the capacitance and energy density of WO_*x*_-based supercapacitors. As a hydrogen evolution reaction (HER) catalyst, WO_*x*_ has a low potential of around − 0.5 V vs. SCE [233]. Reducing pH values of electrolytes could push this limit to − 1 V vs. SCE, but at the cost of cycling stability (Fig. 24e) [234, 241]. Sun et al. achieved both a low cutoff potential to − 0.9 V vs. SCE and good cycling stability with 98% capacitive retained after 30,000 charge–discharge cycles by testing H_*x*_WO_3_ in a mixed electrolyte containing 1 M Na_2_SO_4_ and 0.01 M H_2_SO_4_ (Fig. 24f) [234]. XRD indicated that a new crystal phase (Na_6_WO_6_) formed after cycling in the Na_2_SO_4_ electrolyte (Fig. 24g), creating internal stress and detaching the active material from the current collector (carbon cloth fibers). While cycled in the mixed electrolyte, the structure of H_*x*_WO_3_ was well maintained. It was because, in proton-rich electrolytes, H^+^ insertion and de-insertion became the dominant charge storage mechanism, which was less destructive for the structure of H_*x*_WO_3_. Moreover, the mixed electrolyte enhanced the capacitance of H_*x*_WO_3_, as illustrated by the expanded area enclosed by the CV curve (Fig. 24h). Specifically, when tested in the mixed electrolyte, the electrode exhibited a high areal capacitance of 860 mF cm^−2^, corresponding to 143 F g^−1^, at 5 mV s^−1^ (Fig. 24i). The rational design of electrolyte composition enabled electrodeposited WO_*x*_ electrodes with mutually high capacitance and excellent cycling stability.

### Iron Oxides and Hydroxides

Iron oxides and hydroxides are one of the most attractive, low-cost negative electrode active materials for supercapacitors [242–244]. Electrodeposition is a simple strategy to prepare nanostructured iron oxide and hydroxide [245]. Typically, Fe^2+^ (e.g., Fe(NH_4_)_2_(SO_4_)_2_·6H_2_O) is the iron source [35, 36, 246, 247]. During electrodeposition, Fe^2+^ is first oxidized to Fe^3+^ on positive electrodes, which then combines with OH^−^ that was present in weakly alkaline electrolytes (pH ~ 8) or dissociated from water reduction to form Fe(OH)_3_ deposits. The as-deposited Fe(OH)_3_ further dehydrates to FeOOH in air. The associated chemical reactions are [248]:

11 $${\text{Fe}}^{2 + } \to {\text{Fe}}^{3 + } + e^{ - }$$

12 $${\text{Fe}}^{3 + } + 3{\text{OH}}^{ - } \to {\text{Fe}}({\text{OH}})_{3}$$

13 $${\text{Fe}}({\text{OH}})_{3} \to {\text{FeOOH}} + {\text{H}}_{2} {\text{O}}$$

**Fig. 25:**
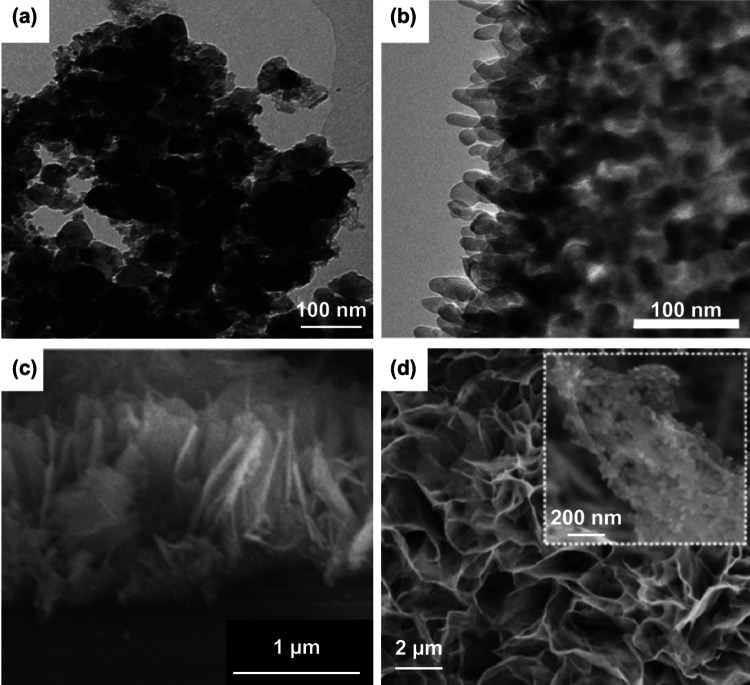
**a**, **b** TEM images of **a** electrodeposited FeOOH nanoparticles and **b** Fe_2_O_3_ nanorods. **c**, **d** SEM images of electrodeposited FeOOH nanosheets and **d** chemically converted Fe_3_O_4_/Fe_2_O_3_ nanosheets. Inset: Magnified view of a nanosheet. Adapted from **a** Ref. [249], **b** Ref. [250], **c** Ref. [251], **d** Ref. [35] with permission

To date, iron oxides of different dimensionalities, including 0D nanoparticles (Fig. 25a) [249], 1D nanorods (Fig. 25b) [250], 2D nanosheets (Fig. 25c) [251], and 3D hierarchical structures (Fig. 25d) [35], have been synthesized electrochemically. For example, Mai et al. reported a cyclic voltammetry method that transformed highly crystalline Fe_2_O_3_ nanoparticles into low-crystalline FeOOH nanoparticles (Fig. 25a) [249]. The resultant FeOOH electrode, with a high mass loading of 9.1 mg cm^−2^, exhibited an outstanding specific capacitance of 716 F g^−1^ at 1 A g^−1^ in 2 M KOH electrolyte. The specific capacitance was attributed to the facilitated ion diffusion kinetics in the FeOOH electrode, but further studies were needed to unveil the mechanism fully.

As electrodeposited iron species are typically Fe(OH)_3_ and FeOOH, thermal conversion to Fe_2_O_3_ with enhanced electrochemical activity is usually performed for processing supercapacitor electrodes [252]. For example, FeOOH nanoneedles were first deposited on Ni-plated ZnO nanorods using a constant potential of 1.5 V vs. Ag/AgCl [253]. During the electrodeposition, the acidic environment created by Fe^3+^ hydrolysis corroded the ZnO nanorods, forming Ni@FeOOH. Subsequent annealing Ni@FeOOH in Ar at 450 °C dehydrated FeOOH to Ni@Fe_2_O_3_ nanoneedles. The Ni nanotubes swiftly conducted electrons between Fe_2_O_3_ and the substrate, while the ultrathin thickness of Fe_2_O_3_ nanosheets endowed fast charge transfer kinetics. Benefiting from these merits, Ni@Fe_2_O_3_ achieved a high specific capacitance of 418 F g^−1^ at 10 mV s^−1^ in the potential window from − 0.8 to 0 V vs. Ag/AgCl, excellent rate capability (215 F g^−1^ at 64 A g^−1^), and cycling stability (93% capacitance retention after 5000 charge–discharge cycles).

In addition to thermal annealing, chemical conversion in hot alkaline solutions represents another means to convert electrodeposited FeOOH into iron oxides. Chemical conversion avoids high-temperature annealing that may trigger structural deformation and particle agglomeration. One excellent example was demonstrated by Sun et al. [35]. They utilized a post-chemical transition method to obtain composite mesoporous iron oxides (Fe_3_O_4_ and Fe_2_O_3_) from electrodeposited FeOOH nanosheets on a 3D exfoliated graphite substrate (EG). First, FeOOH nanosheets were deposited on EG using a potentiostatic method (− 0.5 V vs. SCE for 80 min in an aqueous electrolyte containing 0.05 M Fe(NH_4_)_2_(SO_4_)_2_ and 0.05 M (NH_4_)_2_SO_4_). Afterward, the as-deposited nanosheets were immersed in a 1 M NaOH aqueous solution for 1 h at 70 °C to convert iron oxy-hydroxides to iron oxide (containing Fe_2_O_3_ and Fe_3_O_4_) nanosheets interconnected into a honeycomb-like structure (Fig. 25d). The conversion mechanism is unclear, but we hypothesize that it is associated with dehydration of iron oxy-hydroxides during the treatment. High-resolution SEM image presented that each oxide nanosheet was composed of nanoparticles (Fig. 25d inset). The EG@Fe_3_O_4_/Fe_2_O_3_ electrode delivered an ultrahigh areal capacitance of 1.57 F cm^−2^ at 5 mA cm^−2^, corresponding to 165 F g^−1^ based on the total mass of the electrode. The excellent capacitive performance of the EG@Fe_3_O_4_/Fe_2_O_3_ electrode was ascribed to several factors. First, the hierarchically porous structure with mesopores and macropores in the electrode shortened ion diffusion distance. Second, the heterojunction between Fe_3_O_4_/Fe_2_O_3_ introduced a built-in electric field that facilitated charge transfer between different oxide particles. Third, the interconnected graphene sheets on EG constructed highly conductive electron transport networks that minimized capacitance loss at fast discharging rates.

### Nickel/Cobalt Oxides and Hydroxides

A critical issue that we would like to highlight first in this section is the concepts of intrinsic and extrinsic pseudocapacitive materials. Intrinsic pseudocapacitive materials refer to materials that always show pseudocapacitive characteristics (CV curves with broad or no redox peaks and plateau-free charge–discharge profiles) irrespective of size. Extrinsic pseudocapacitive materials exhibit pseudocapacitive signatures only at nanoscales. This transition of the electrochemical behaviors of extrinsic pseudocapacitive materials was due to the reduced ion diffusion distance that accelerated charge storage kinetics and, in some cases, suppressed phase transformations [13].

**Fig. 26:**
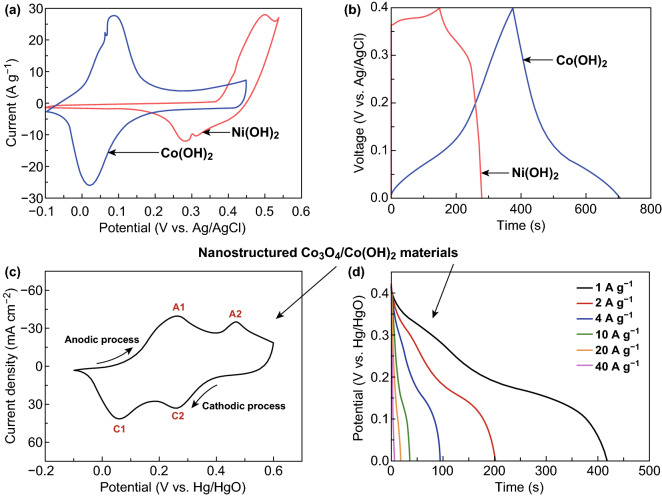
**a** CV curves and **b** galvanostatic charge–discharge profiles of Co(OH)_2_ (blue) and Ni(OH)_2_ (red). **c** CV curves and **d** galvanostatic charge–discharge profiles of Co_3_O_4_/Co(OH)_2_ core/shell nanowire arrays. Adapted from **a**, **b** Ref. [255], **c**, **d** Ref. [267] with permission

Nickel and cobalt oxides and hydroxides are extrinsic pseudocapacitive materials [254]. Charge storage in their bulk forms involves phase transitions, resulting in apparent redox peaks in CV curves (Fig. 26a) and plateaus in galvanostatic charge–discharge profiles (Fig. 26b) [255]. Therefore, these battery-like materials have been used as electrodes in aqueous batteries [256–258] or battery-type electrodes in hybrid supercapacitors [96, 259–261], but cannot be studied in the context of pseudocapacitors. However, once their size shrinks to nanoscales, their pseudocapacitive features will emerge [262–266], as indicated by the broad redox peaks in CV curves (Fig. 26c) and sloping charge–discharge profiles (Fig. 26d) [267].

Nickel and cobalt (hydro)oxides are electrochemically synthesized via cathodic deposition in aqueous solutions. Ni^2+^ or Co^2+^ combines with OH^−^ produced from water or anions in electrolytes. Taking Co(OH)_2_ as an example, one reported electrodeposition mechanism in Co(NO_3_)_2_ aqueous solutions is [268]:

14 $${\text{NO}}_{3}^{ - } + {\text{H}}_{2} {\text{O }} + 2e^{ - } \to {\text{NO}}_{2}^{ - } + 2{\text{OH}}^{ - }$$

15 $${\text{Co}}^{2 + } + 2{\text{OH}}^{ - } \to {\text{Co}}({\text{OH}})_{2}$$

**Fig. 27:**
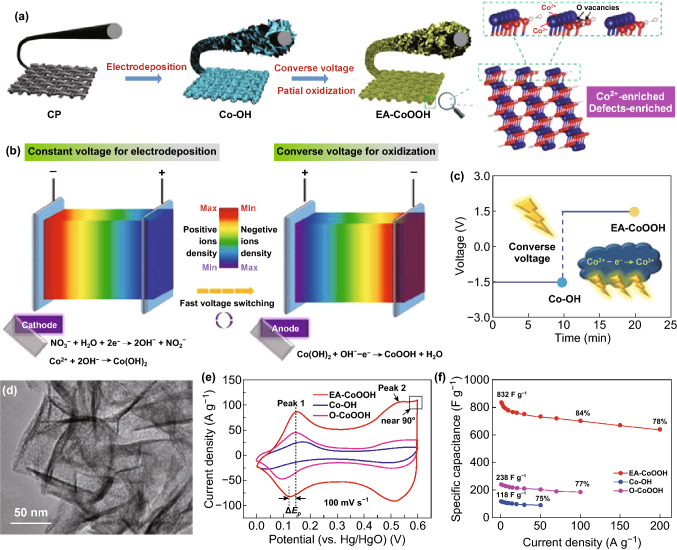
**a** Schemes of the synthesis steps of EA-CoOOH by a converse voltage method and the molecular structure of Co^2+^- and defect-rich EA-CoOOH. **b** Schemes showing the ion concentration gradients during constant voltage for electrodeposition (left) and converse voltage for oxidization (right). **c** Voltage profiles of electrodeposition and converse voltage stages. **d** TEM image of EA–CoOOH. **e** CV curves and **g** rate capability of EA–CoOOH in comparison with other electrodes. Adapted from Ref. [268] with permission

Guo et al. have recently demonstrated a converse voltage strategy to activate electrodeposited Co(OH)_2_ nanosheets on carbon fibers (Fig. 27a) [268]. First, Co(OH)_2_ nanosheets were electrodeposited with a constant potential of − 1.5 V vs. SCE (Fig. 27b). The voltage then reversed (1.5 V vs. SCE) (Fig. 27c), triggering a phase transition from Co(OH)_2_ to low-crystalline CoOOH (denoted as EA-CoOOH) containing abundant structural defects (oxygen vacancies, lattice disorders, and interconnected mesopores) (Fig. 27d). Quantitative analyses on the electrode kinetics revealed that the capacitance of EA-CoOOH contributing from kinetically fast, surface-controlled processes occupied 93% at 5 mV s^−1^, and further increased to 99% at 100 mV s^−1^. The nanosheets and defects altered the intrinsic battery-type behavior of CoOOH to pseudocapacitive characteristics. Outstandingly, EA–CoOOH electrode exhibited substantially enhanced capacitance than Co(OH)_2_ and deeply oxidized O–CoOOH electrode (Fig. 27e), delivering a high specific capacitance of 832 F g^−1^ at 1 A g^−1^ and retained 78% of the capacitance (649 F g^−1^) at 200 A g^−1^ (Fig. 27f).

Introducing a secondary cation, such as Fe^3+^ and Al^3+^, in Ni/Co hydroxides to form double hydroxides have been proved to enhance capacitive performance due to charge hopping or valence interchange between different cations [96, 266, 269, 270]. Double hydroxides usually possess layered structures with large interlayer spacings (e.g., ~ 1 nm), which resulted in their high specific capacitance (e.g., > 2000 F g^−1^) [96, 106, 271]. Unfortunately, their limited potential windows in alkaline electrolytes (~ 0.5 V) and poor rate performance still hinder their large-scale applications in aqueous batteries and supercapacitors. Neutral aqueous electrolytes can suppress appreciable oxygen evolution in alkaline solutions and thus extend the potential window of layered double hydroxides (LDHs) to ~ 1 V.

**Fig. 28:**
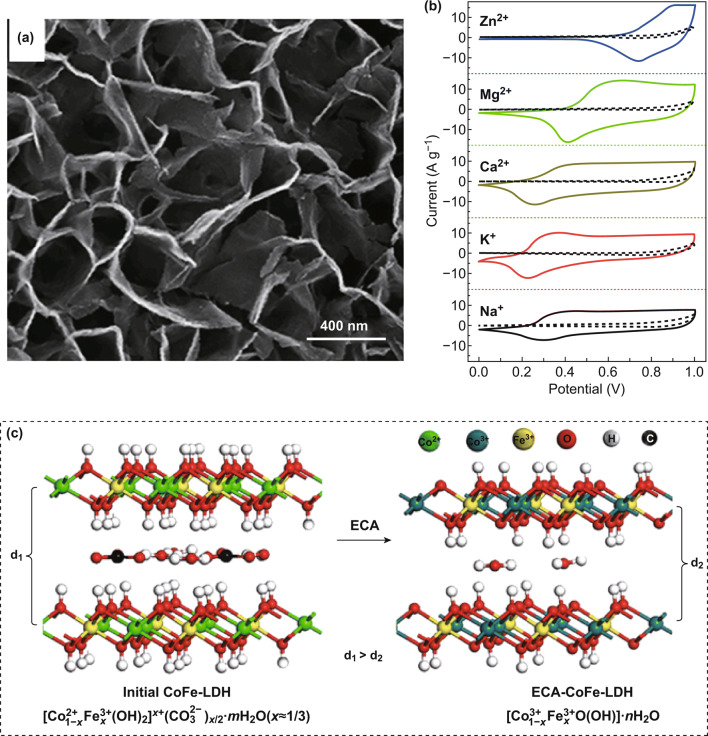
**a** SEM image of CoFe layered double hydroxide (LDH) nanoplates. **b** CV curves and **c** galvanostatic charge–discharge profiles of CoFe-LDH in different aqueous solutions. **d** Evolution of the crystal structure and composition of CoFe-LDH before and after electrochemical activation. **e** Adsorption energies of various metal ions over H-terminated (magenta) and O-terminated (blue) LDH laminates. Reproduced from Ref. [272] with permission

Since anions residing between the interlayer space and the electrostatic repulsion from positively charge LDH laminate, cation intercalation into LDHs in neutral electrolytes remains thermodynamically unfavorable, resulting in unsatisfactory electrochemical performance. Recently, Li et al. have demonstrated an electrochemical strategy to ease the cation intercalation into LDHs in neutral electrolytes [272]. Co–Fe LDH nanoplates were first electrodeposited on Ni foam using a cathodic electrodeposition method (Fig. 28a). Subsequently, electrochemical activation (EA) of the Co–Fe LDH nanosheets was conducted by CV between 0 and 0.6 V vs. SCE in KOH or NaOH electrolyte (denoted as EA–Co–Fe LDH). The obtained electrode exhibited pronounced electrochemical activity in various aqueous electrolytes, including NaNO_3_, KNO_3_, Ca(NO_3_)_2_, Mg(NO_3_)_2_, Zn(NO_3_)_2_ between 0 and 1 V vs. SCE (Fig. 28b, c). The specific capacitance of EA–Co–Fe LDH in NaNO_3_ reached 417 F g^−1^, which was 27 times higher than that of as-deposited Co–Fe LDH nanoplates. XRD, XPS, FTIR, and X-ray absorption near-edge structure (XANES) all indicated that during the electrochemical activation, Co(OH)_2_ in CoFe-LDH was oxidized to CoOOH, resulting in hydrogen vacancies and removal of carbonate anions residing within the interlayer space of CoFe-LDH (Fig. 28d). Density functional theory calculations elucidated the adsorption sites of metal ions in LDH and EA-LDH materials (Fig. 28e). The results suggested that the adsorption of metal ions (Li^+^, Na^+^, K^+^, Ca^2+^, Mg^2+^, and Zn^2+^) on terminal H was unfavorable, whereas adsorptions of the same set of ions on O termination were thermodynamically stable, as reflected from the negative adsorption energy (Fig. 28e, blue bars). The enhanced adsorption tendency with O terminals was ascribed to the H vacancies formed during the activation step. Taking together, the formation of H vacancies and extraction of interlayer anions after the electrochemical activation together imparted the ion intercalation capability of CoFe-LDH in neutral electrolytes.

## Composites

Composites that combine the merits of two or more materials are versatile electrode candidates for supercapacitor electrodes. These composites are typically electrodeposited through a one-step co-electrodeposition. The reported electrodeposited composites are classified according to their constituent species, including composites having the same group of materials, such as oxide/oxide (manganese oxide/molybdenum oxide [273, 274], nickel manganese oxide [275], molybdenum oxide/tungsten oxide [276]), and composites with different types of materials, including oxide/conducting polymer (polypyrrole/manganese oxide [277, 278], manganese oxide/polyaniline [279, 280], manganese oxide/poly(3,4-ethylenedioxythiophene):poly(styrenesulfonate) [281, 282], polyaniline/vanadium oxide [27], polypyrrole/vanadium oxide [283, 284], polypyrrole/molybdenum oxide [285], polyaniline/tungsten oxide [286]) and oxide/hydroxide (NiAl-layered double hydroxide/manganese oxide [287] and manganese oxide/nickel hydroxide [288]). Their capacitance and corresponding synthesis methods are summarized in Table 2. Electrodeposited composites usually exhibit advantages of synergies and strong interactions among the incorporated materials that exhibit superior capacitive performance to the corresponding single-component counterparts.

**Table 2:** Different types of composites synthesized via electrochemical methods

Component #1	Component #2	Synthesis method	Capacitance	References
*Conducting polymer–metal oxide*				
Polypyrrole	MnO_*x*_	Galvanostatic deposition	463 F g^−1^@2 A g^−1^	[277]
Polyaniline	MnO_*x*_	Cyclic voltammetry Pulse potential	415 F g^−1^@1.67 mA cm^−2^ 90.25 F g^−1^@1 mA g^−1^	[279] [280]
Poly(3,4-ethylenedioxythiophene):poly(styrenesulfonate)	MnO_*x*_	Constant potential Constant potential	1.67 F cm^−2^@0.5 mA cm^−2^ 386 mF cm^−2^@1 mA cm^−2^	[281] [282]
Polyaniline	VO_*x*_	Cyclic voltammetry	0.66 F cm^−2^@0.5 mA cm^−2^	[27]
Polypyrrole	VO_*x*_	Constant potential Constant potential	750 F g^−1^@5 A g^−1^ 412 F g^−1^@4.5 mA cm^−2^	[283] [284]
Polypyrrole	MoO_*x*_	Cyclic voltammetry	398 F g^−1^@1 A g^−1^	[285]
Polyaniline	WO_*x*_	Cyclic voltammetry	408 F g^−1^@1 A g^−1^	[286]
*Metal oxide–metal oxide*				
MnO_*x*_	MoO_*x*_	Cyclic voltammetry Galvanostatic deposition	21 mF cm^−2^@50 mV s^−1^ 408 F g^−1^@2 mV s^−1^	[273] [274]
NiMnO_*x*_		Cyclic voltammetry	961.5 F g^−1^@10 mV s^−1^	[275]
MoO_*x*_	WO_*x*_	Galvanostatic deposition	517.4 F g^−1^@1 A g^−1^	[276]
*Hydroxide–metal oxide*				
NiAl-layered double hydroxide	MnO_*x*_	Constant potential	1554 F g^−1^@1 A g^−1^	[287]
Nickel hydroxide	MnO_*x*_	Constant potential	344 F g^−1^@0.5 A g^−1^	[288]

Not all the active materials are co-electrodepositable in one step. One prerequisite is that the materials to be compounded must be synthesizable under similar electrochemical conditions, including pH value, polarization potential, current density, and temperature. For example, Zou et al. electrodeposited a tungsten oxide and polyaniline composite via one-step cyclic voltammetry in a mixed acidic electrolyte containing tungstic acid and aniline monomers [289]. The success of the co-deposition was owing to the acidic environment needed for both tungsten oxide and polyaniline. In contrast to the densely stacked tungsten oxide particles, the composite possessed widened pores and interconnected particles. This difference in the morphology was ascribed to the incorporated polyaniline, which acted as a structural scaffold that prevented the aggregation of tungsten oxide particles. The composite electrode exhibited enhanced pseudocapacitive performance over a wide potential window from − 0.5 to 0.7 vs. SCE, which combined the potential windows of polyaniline (− 0.1–0.7 V vs. SCE) and tungsten oxide (− 0.5–0.3 V vs. SCE). The synergistic effects between polyaniline and tungsten oxide improved the capacitive performance of the composite. Tungsten oxide offered high pseudocapacitance. Polyaniline created interparticle pores that increased surface area and conducted electrons to maintain superior rate capability to those of bare tungsten oxide or polyaniline.

**Fig. 29:**
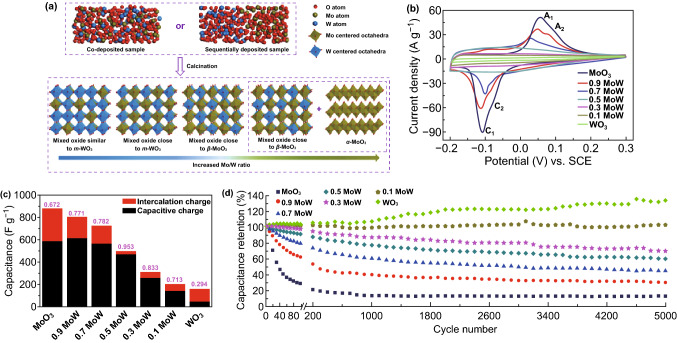
**a** Schemes of the formation mechanism and crystal structure of Mo–W mixed oxides. **b** CV curves, **c** capacitance contributions (capacitive vs. diffusion-controlled processes), **d** cycling stability, and **e** ion transport patterns of different Mo–W mixed oxides. Reproduced from Ref. [276] with permission

Another example is a series of molybdenum–tungsten mixed oxide composites co-electrodeposited into titanium dioxide nanotube arrays using a one-step galvanostatic plating [276]. The plating electrolyte was an aqueous mixture of different concentrations of sodium molybdate dihydrate, sodium tungstate dihydrate, ethylenediamine tetra-acetic acid disodium, and ammonium acetate. The as-electrodeposited amorphous Mo–W mixed oxide film was thermally annealed at 450 °C in air to improve its crystallinity. The similarities in the valence state, ionic radius, and electronegativity of W^6+^ and Mo^6+^ were the keys to the successful co-deposition. XRD and Raman spectroscopy indicated that the fabricated Mo–W mixed oxides were monoclinic, and the crystal structure transited from *m*-WO_3_-like to *β*-MoO_3_-like when increasing the Mo/W ratio (Fig. 29a). Though pure MoO_3_ exhibited higher capacitance than the composite electrodes at 20 mV s^−1^ (Fig. 29b), its rate capability (Fig. 29c) and cycling stability (Fig. 29d) were inferior to those of the composites. Among all the synthesized Mo–W mixed oxides, 0.5MoW with a Mo/W ratio of 1 achieved the best electrochemical performance: high specific capacitance of 517.4 F g^−1^ at 1 A g^−1^ and good capacitance retention of 89.3% at 10 A g^−1^. The enhanced rate capability of the composites over that of MoO_3_ was attributed to two factors. First, the long-range ordered structure of pure oxides was disrupted in the mixed oxides, which decreased crystal size, increased surface area, and facilitated ion diffusion. Second, the disordered monoclinic crystal structure of the mixed oxides yielded a larger lattice space than those of WO_3_ and MoO_3_ alone, resulting in decreased ion diffusion resistance.

**Fig. 30:**
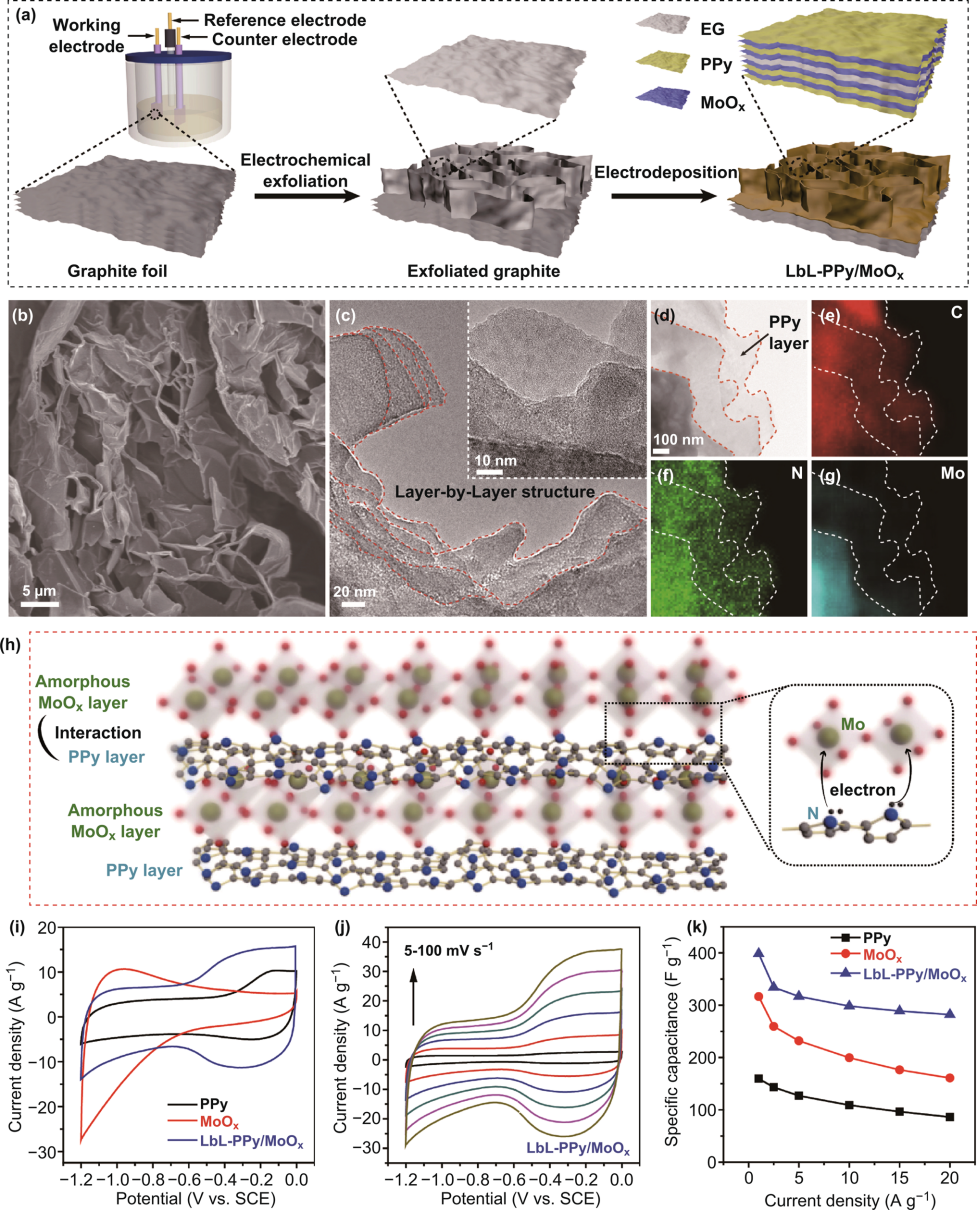
**a** Schemes of the synthesis steps, **b** SEM image, **c** TEM image, and **d-g** elemental mappings of layer-by-layer PPy/MoO_*x*_. **h** Interactions between PPy and MoO_*x*_ layers. **i** CV curves of PPy, MoO_*x*_, and PPy/MoO_*x*_. **j** CV curves of PPy/MoO_*x*_ at different scan rates. **k** Rate capability of PPy, MoO_*x*_, and PPy/MoO_*x*_. Reproduced from Ref. [285] with permission

Recently, Zhang et al. have demonstrated a facile cyclic voltammetry method capable of depositing coupled strongly, layer-by-layer PPy/MoO_*x*_ composite films on 3D exfoliated graphite substrates (Fig. 30a) [285]. MoO_*x*_ layer was first deposited on graphite foil during the cathodic scan, while the PPy layer was subsequently grown on MoO_*x*_ in the following anodic scan. Therefore, a layer-by-layer PPy/MoO_*x*_ structure was obtained after multiple depositions (Fig. 30b–d). In addition, Fourier transform infrared spectroscopy and X-ray photoelectron spectroscopy detected the reduction in Mo valence and the enhancement of protonation level of PPy, both of which resulted from the strong coupling between PPy and MoO_*x*_ (Fig. 30h) that are beneficial for enhancing the electrochemical performance. The composite electrode exhibited a specific capacitance of 398 F g^−1^ at 1 A g^−1^, higher than those of PPy (160 F g^−1^) and MoO_*x*_ (320 F g^−1^) at identical current density (Fig. 30i–k).

**Fig. 31:**
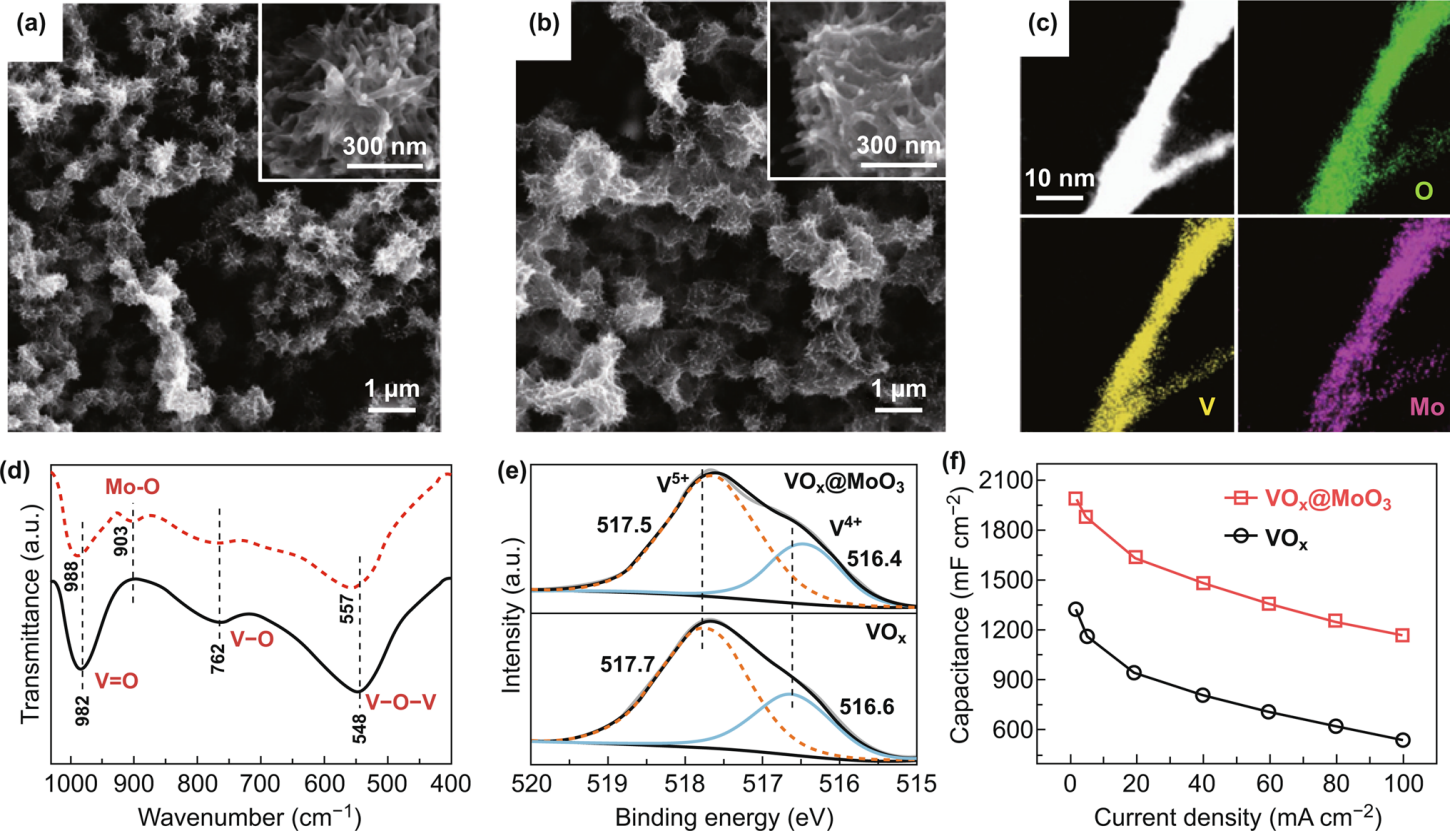
**a**, **b** SEM images of **a** VO_*x*_ and **b** VO_*x*_@MoO_3_. **c** Elemental mappings of VO_*x*_@MoO_3_. **d** FTIR spectra of VO_*x*_ and VO_*x*_@MoO_3_. **e** V 2*p*_3/2_ XPS spectra of VO_*x*_ and VO_*x*_@MoO_3_. **f** Areal capacitances of VO_*x*_ and VO_*x*_@MoO_3_ as a function of current density. Reproduced from Ref. [295] with permission

Besides one-step co-electrodeposition, composites have been prepared using multistep electrodeposition methods [290–294]. For example, Wang and Cai et al. synthesized a VO_*x*_@MoO_3_ composite through a two-step electrochemical deposition. VO_*x*_ nanorods were first grown on carbon cloth by cyclic voltammetry (Fig. 31a) [295]. Subsequently, a thin layer of MoO_3_ was coated on the VO_*x*_ nanorods by a constant current deposition for 9 s (Fig. 31b, c). The composite electrode VO_*x*_@MoO_3_ displayed an areal capacitance of 1980 mF cm^−2^ at 2 mA cm^−2^, against 1309 mF cm^−2^ of VO_*x*_ and 233 mF cm^−2^ of MoO_3_ under identical testing conditions. Fourier transform infrared spectroscopy revealed that the V–O–V peaks of VO_*x*_@MoO_3_ blueshifted in comparison with that of VO_*x*_ (Fig. 31d). The V 2*p*_3/2_ XPS spectrum showed that the binding energies of both V^5+^ and V^4+^ of VO_*x*_@MoO_3_ downshifted by 0.2 eV. Both results indicated that the electronic structure and chemical environment of VO_*x*_ in VO_*x*_@MoO_3_ were modified by the strong interaction between VO_*x*_ and MoO_3_, accounting for the improved capacitive performance. Specifically, VO_*x*_@MoO_3_ reached a high areal capacitance of 1980 mF cm^−2^ at 2 mA cm^−2^, exceeding 1309 mF cm^−2^ of VO_*x*_ (Fig. 31f). Strong interaction among the incorporated materials is indispensable in realizing the synergy of the composite electrodes. Poor interactions will not only interrupt electron transport but also induce structural instability of composite [296].

## Other Materials

Electrodeposition has occasionally been used to synthesize nano-/microstructured sulfides [297–300], polyanionic compounds [28], and selenides [301]. These materials typically exhibit higher capacitance than their corresponding oxides due to the different chemical environments created by substituting oxygen near the redox sites, which enhanced electrical conductivity or led to crystal structures favorable for fast ion diffusion, e.g., channels, slits, and pores [28, 297, 302, 303]. Electrochemical syntheses of these materials usually proceed in electrolytes containing precursors of sulfide, phosphate, or selenide.

Using a one-step electrochemical co-deposition method, Chen et al. electrodeposited ternary nickel–cobalt sulfide nanosheets on carbon cloth [302]. The electrodeposition was conducted in a three-electrode electrolytic cell containing 5 mM CoCl_2_ with different concentrations of NiCl_2_ (1, 2.5, 5, 7.5, and 10 mM) and 0.75 M thiourea. The deposition technique was cyclic voltammetry within a potential range between − 1.2 and 0.2 V vs. Ag/AgCl at 5 mV s^−1^ for 15 cycles. SEM displayed that the electrodeposited materials formed a dense array of highly porous nanosheets. High-angel annular dark-field scanning transmission electron microscopy (HAADF-STEM) and energy-dispersive X-ray spectroscopy (EDS) confirmed the uniform distributions of Co, Ni, and S in the deposited nanosheets. The as-deposited sulfide exhibited extrinsic pseudocapacitive characteristics of symmetric and plateau-free charge–discharge profiles. The sulfide with an optimal Ni-to-Co ratio (Ni–Co–S-4) showed the highest specific capacitance of 1418 F g^−1^ at 5 A g^−1^ among all Ni–Co–S compounds. It retained 90.6% of its initial capacitance when the current density increased to 100 A g^−1^. Falola et al. synthesized MoS_2_ film on a glass carbon electrode using an electrochemical deposition coupled with thermal annealing [300]. The electrochemical deposition was conducted via a CV scan from − 1.2 to 1 V vs. Ag/AgCl in an aqueous electrolyte containing 10 mM (NH_4_)_2_MoS_4_ and 0.2 M KCl at pH = 6.8, following the reaction [304]:

16 $${\text{MoS}}_{4}^{2 - } + 4{\text{H}}^{ + } + 2e^{ - } \to {\text{MoS}}_{2} + 2{\text{H}}_{2} {\text{S}}$$

The as-deposited MoS_2_ was thermally annealed under Ar at 600 °C to increase its crystallinity. The resultant MoS_2_ film exhibited a gravimetric capacitance of ~ 500 F g^−1^ at 0.75 A g^−1^. Increasing the film thickness from 50 to 200 nm decreased the capacitance to ~ 100 F g^−1^, which could be due to the sluggish ion diffusion kinetics throughout the thick films.

**Fig. 32:**
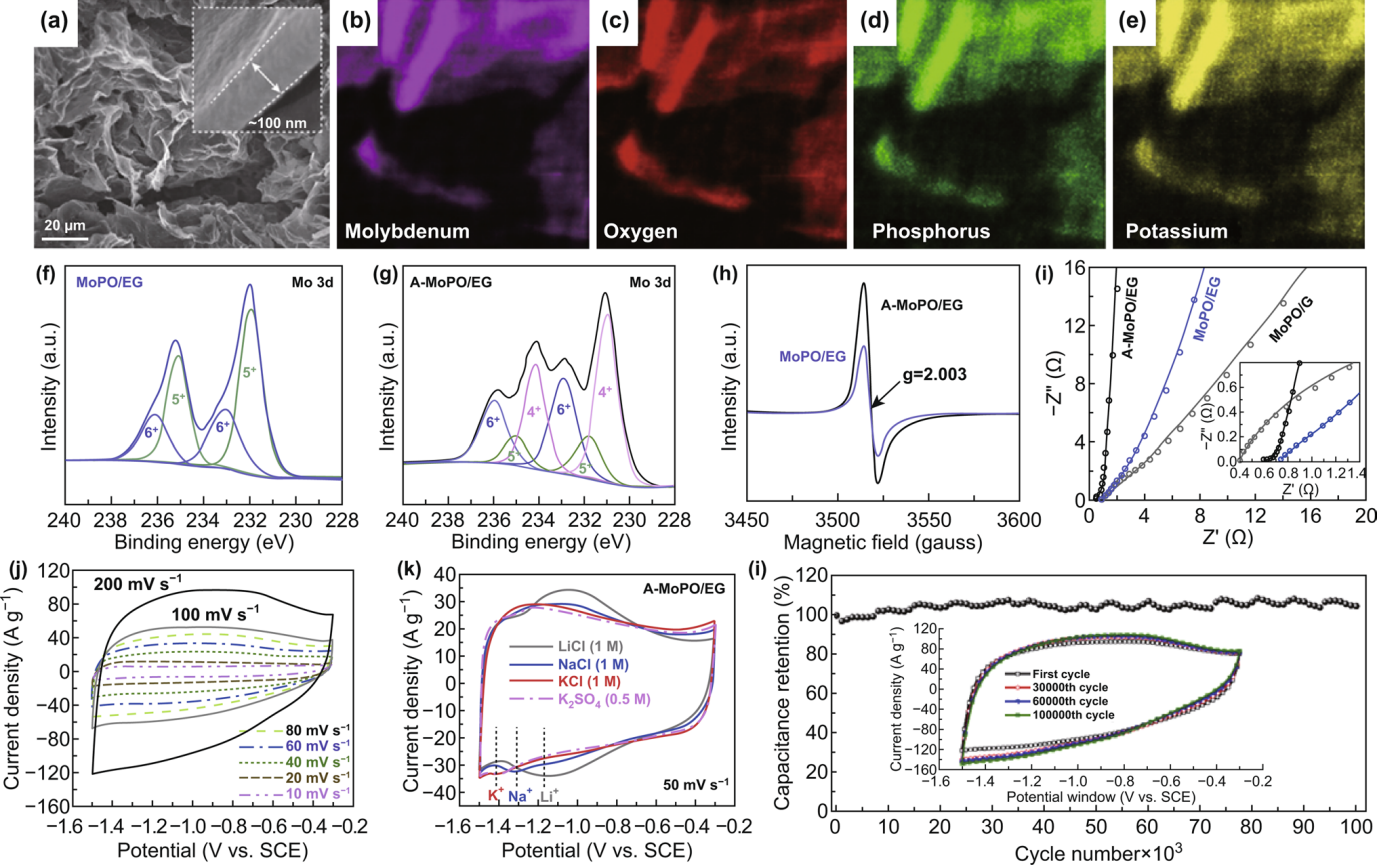
**a** SEM image of activated polyanionic molybdenophosphate (A-MoPO/EG) on electrochemically exfoliated graphite foil. Inset: Magnified view showing the thickness of A-MoPO. **b**–**e** EDS elemental mappings of Mo, O, P, and K in A-MoPO/EG. **f**, **g** Mo 3d XPS spectra of **f** MoOP/EG and **g** A-MoPO/EG. **h** EPR spectra of oxygen deficiency signals of MoOP/EG and A-MoOP/EG. **i** Nyquist plots of A-MoPO/EG, MoPO/EG, and MoPO/non-exfoliated graphite foil electrodes. **j** CV curves of A-MoPO/EG at different scan rates in 3 M aqueous KCl electrolyte. **k** CV curves of MoPO/EG recorded in various aqueous electrolytes. **i** Cycling stability of A-MoPO/EG in 100,000 charge–discharge cycles. Adapted from Ref. [28] with permission

Molybdenophosphate (A–Mo–O–P, A = Na, K, etc.) materials are a family of polyanionic phosphate compounds with open frameworks, which have recently aroused great interest in rechargeable batteries [305–308]. Similar promising performance is also expected in capacitive applications, albeit seldomly reported. Recently, Song et al. have electrosynthesized a polyanionic molybdenophosphate film on a 3D exfoliated graphite substrate (EG) using a galvanostatic method [28]. The deposition electrolyte was 0.025 M ammonium molybdate mixed with 0.2 M phosphate buffer. The polyanion PO_4_^3−^ in the electrolyte were incorporated into the mixed-valence Mo oxide lattice (containing Mo^5+^ and Mo^6+^), replacing O atoms and forming Mo–O–P bonds (denoted as MoPO/EG). MoPO/EG was in situ electrochemically activated in 3 M KCl using cyclic voltammetry for 10,000 cycles at a scan rate of 200 mV s^−1^ (denoted as A-MoPO/EG). This process allowed repeated K^+^ intercalation and de-intercalation. A-MoPO/EG exhibited a film morphology with an average thickness of 100 nm (Fig. 32a). EDS indicated the even contributions of Mo, O, P, and K elements in A-MoPO/EG (Fig. 32b–e). The stable P signal after the activation indicated the electrochemical stability of Mo–O–P bonds. Inductively coupled plasma mass spectroscopy (ICP-OES) suggested the chemical formulae of MoPO and A-MoPO were K_0.7_Na_0.35_Mo_2_O_4.5_PO_4_ and K_1.55_Mo_2_O_4.2_PO_4_, respectively. Due to the incorporation of Mo^4+^ after the activation, the average valence state of Mo in MoPO/EG was reduced from + 5.38 to + 4.84 (Fig. 32f, g). Meanwhile, the content of O vacancy increased, as indicated by the enhanced intensity in the electron spin resonance (EPR) spectra (Fig. 32h). Additionally, all Na^+^ in MoPO/EG was fully exchanged by K^+^ and additional K^+^ incorporated, both expanding the lattice spacing of molybdophosphate. Benefiting from the widened layers, as well as the enhanced electrical conductivity brought by the O vacancy, A-MoPO/EG showed better charge transfer kinetics than MoPO/EG (Fig. 32i). A-MoPO/EG exhibited quasi-rectangular CV curves, even at scan rates up to 200 mV s^−1^ (Fig. 32j). CV (Fig. 32k), XPS, and inductively coupled plasma mass spectroscopy (ICP-MS) indicated that cation (e.g., Li^+^, Na^+^, and K^+^) intercalation was the primary charge storage mechanism of A-MoPO/EG. This polyanionic negative electrode exhibited a high specific capacitance of 556 F g^−1^ at 4.5 A g^−1^, a low cutoff potential window limit of − 1.5 V vs. SCE, as well as high electrochemical durability without capacitance decay after 100,000 charge–discharge cycles (Fig. 32l). The authors speculated that the stable potential window of A-MoPO/EG down to − 1.5 V vs. SCE could be due to the reversible K^+^-intercalation (around − 1.4 V vs. SCE) near the water splitting potential, which suppressed hydrogen gas evolution [309].

Besides electrodeposition, electrochemical exfoliation is applicable to synthesize high-quality two-dimensional (2D) nanomaterials, e.g., MoS_2_, boron nitride, and MXene, from their corresponding bulk materials [310]. One example is 2D titanium carbide, Ti_3_C_2_T_*x*_ (T = O and OH), belonging to the MXene family [311]. Yang et al. applied a constant potential of 5 V for 5 h that delaminated bulk TiAlC_2_ into Ti_3_C_2_T_*x*_. The experimental setup was a two-electrode system with the first exfoliation step in an aqueous electrolyte composed of 1.0 M ammonium chloride (NH_4_Cl) and 0.2 M tetramethylammonium hydroxide (TMA·OH). The subsequent delamination in 25 wt % TMA·OH yielded single- or double-layer Ti_3_C_2_T_*x*_ flakes with sizes up to 18.6 μm. During the electrochemical etching, Cl^−^ ions etched Al and broke the Ti–Al bonds. The intercalation of ammonium hydroxide subsequently opened the edges of the etched materials and triggered the etching. This fluoride-free electrochemical exfoliation process provided a safe and scalable way to synthesize MXenes. As a supercapacitor electrode, the Ti_3_C_2_T_*x*_ film exhibited an areal capacitance of 220 mF cm^−2^ (volumetric capacitance 439 F cm^−3^) at 10 mV s^−1^, comparable or even superior to those made from traditional wet chemical etching methods.

## Summary and Outlook

**Fig. 33:**
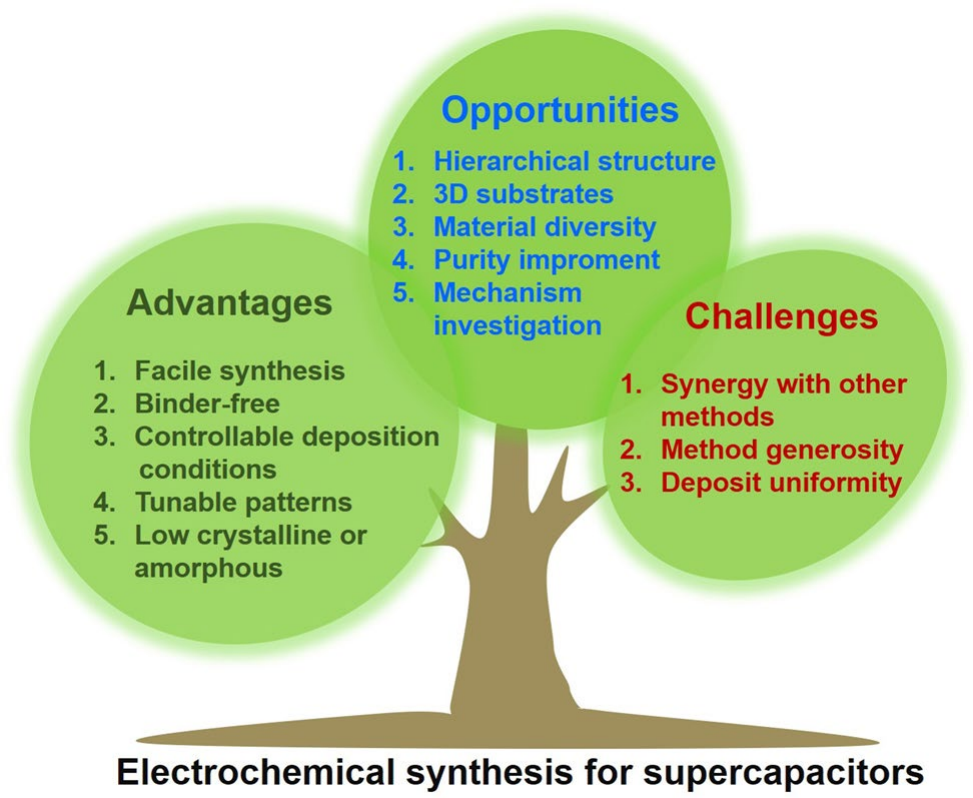
Advantages, challenges, and future opportunities of electrochemical synthesis for supercapacitors

Electrochemical methods constitute a family of facile, economic, and versatile synthesis technology for a plethora of nano-/microstructured materials as active materials in supercapacitors. It has its unique advantages, challenges, and opportunities as a synthesis tool (Fig. 33). In this review article, we have demonstrated that electrodeposition strategies can synthesize active materials consisting of zero-dimensional, one-dimensional, two-dimensional, and three-dimensional nano-/micromaterials, including particles, rods, tubes, wires, plates, sheets, and hierarchical structures.

To sum up, we have listed below the advantages of electrochemical technologies in preparing nano-/microstructured materials for supercapacitors or, broadly speaking, electrochemical energy storage devices.

1. Their synthesis conditions are often mild (e.g., under room temperature) without elevated temperatures or ultrahigh pressures that might damage the structural integrity or alter the composition of the deposits and substrates. Additionally, electrochemical synthesis requires no advanced instruments and sophisticated operations, making it highly attainable and readily achievable.
2. Electrodeposition directly and seamlessly incorporates active materials onto current collectors, a feature eliminating the need for polymer binders and conductive additives and easing electrode preparations.
3. Electrodeposition offers facile tunability over the composition, crystal phase, and morphology of the deposited materials via changing depositing conditions, electrolyte compositions, current, voltage, and temperature.
4. Electrodeposition is possible to direct the growth of active components onto user-designed patterns, as it only deposits materials in electrically conductive regions. This deposition selectivity makes electrodeposition particularly suitable to coat active materials on supercapacitors with delicate electrode architectures, such as micro-supercapacitors with interdigitated electrodes [196, 312, 313]. Additionally, the preferred deposition at ion-accessible locations ensures the high utilization efficiency of the active materials.
5. Electrochemically synthesized materials are usually poorly crystalline or completely amorphous. The amorphous nature and abundant defects sometimes are beneficial for enhancing capacitance.

Despite the above strengths, electrochemical techniques are facing many challenges and difficulties, including:

1. Post-treatments after electrodeposition, which are usually needed to improve the crystallinities of deposits, might alter the mechanical strength, functionality concentrations, crystal structures, or porous structure. For example, electrochemically exfoliated graphene sheets are usually stacks of 2–20 nm thick, instead of few- or single-layer graphene. Therefore, ultrasonication is needed to delaminate the exfoliated graphene into few-layer graphene sheets. This process, however, will inevitably break the resultant graphene sheets into pieces that will increase sheet-to-sheet contact resistance.
2. As discussed in Sect. 7, some active materials, e.g., metal sulfides, have not been extensively synthesized directly by electrodeposition. Converting metal oxides into the corresponding sulfides through sulfurization is one possible way. Still, this post-conversion usually involves high temperatures that might encounter problems with thermally unstable compounds or loss of structural water that are critical for charge transport [314].
3. Controlling the uniformity of deposits remains a grand hurdle for electrodeposition, as edges of the deposition substrates are usually deposited first due to the strong local electrical field.

Projecting forward, we believe the following issues, if adequately addressed, could considerably enhance the electrochemical performance of electrodeposited materials and the practicality of electrodeposition.

1. Rational design and realization of hierarchical structures with one-step electrodepositions is highly attractive to make high-performance supercapacitor electrodes with high mass loadings. Alternatively, combining electrodeposition strategies with other established materials synthesis methods (e.g., hydrothermal reactions to induce Ostwald ripening [197]) could also be explored to achieve hierarchical structures.
2. Developing substrates with mutually high surface areas and excellent electrical conductivity is preferred to improve the ion diffusion kinetics in supercapacitors, but one must be meticulous about the deposition time and rate to avoid pore clogging. In this respect, self-limiting electrodeposition to control the deposit thickness is necessary [315].
3. Diversifying the materials synthesized by electrodeposition. For example, metal sulfides have gained increasing attention as a new generation of electrochemical energy storage materials [316–318], but it is a pity that electrochemical methods can hardly synthesize them without post-treatments. Besides metal sulfides, electrodeposition can grow electrochemically active, 2D materials beyond graphene (e.g., layered metal oxides/hydroxides, boron nitride, and *g*-C_3_N_4_) [319], porous polymers and organic compounds, or pseudocapacitive materials compatible with non-aqueous electrolytes. The room for the development of electrochemical synthesis technologies is undoubtedly immense.
4. The purity of electrodeposited materials is often subpar to those made by vapor phase depositions. The multivalence could benefit charge storage, but might pose challenges for studies on charge storage mechanisms because differentiating outcomes resulting from collective behaviors of all the active components is non-trivial.
5. Deepening the understandings of the electrodepositions mechanism, including electrochemical nucleation and growth processes, will be highly rewarding to propel the development of electrochemical synthesis. For example, understanding the microscopic, complicated, and transient nucleation processes is imperative for controlling the morphology and uniformity of deposits. Mechanistic studies of electrodeposition, especially those developed recently, are limited. With the aid of in situ imaging technologies practiced in battery communities, the gain of insights into the early stages of electrodeposition will accumulate steadily.

Finally, we would like to stress that electrochemical synthesis technologies by no means can replace any existing materials synthesis methods. On the contrary, electrochemical strategies need to cooperate with other techniques to facilitate the explorations and development of high-performance electrodes in supercapacitors. Considering that electrodes play a central role in charge storage performances, we envision that the advancement and diversification of electrochemical synthesis technologies will directly push the development of electrochemical energy storage devices within and beyond electrochemical energy storage fields.
